# TOR complex 2 contributes to regulation of gene expression via inhibiting Gcn5 recruitment to subtelomeric and DNA replication stress genes

**DOI:** 10.1371/journal.pgen.1010061

**Published:** 2022-02-14

**Authors:** Adiel Cohen, Emese Pataki, Martin Kupiec, Ronit Weisman

**Affiliations:** 1 Department of Natural and Life Sciences, The Open University of Israel, Ra’anana, Israel; 2 The Shmunis School of Biomedicine & Cancer Research, Tel Aviv University, Tel Aviv, Israel; University of Ottawa, CANADA

## Abstract

The fission yeast TOR complex 2 (TORC2) is required for gene silencing at subtelomeric regions and for the induction of gene transcription in response to DNA replication stress. Thus, TORC2 affects transcription regulation both negatively and positively. Whether these two TORC2-dependent functions share a common molecular mechanism is currently unknown. Here, we show that Gad8 physically interacts with proteins that regulate transcription, including subunits of the Spt-Ada-Gcn5-acetyltransferase (SAGA) complex and the BET bromodomain protein Bdf2. We demonstrate that in the absence of TORC2, Gcn5, the histone acetyltransferase subunit of SAGA, accumulates at subtelomeric genes and at non-induced promoters of DNA replication genes. Remarkably, the loss of Gcn5 in TORC2 mutant cells restores gene silencing as well as transcriptional induction in response to DNA replication stress. Loss of Bdf2 alleviates excess of Gcn5 binding in TORC2 mutant cells and also rescues the aberrant regulation of transcription in these cells. Furthermore, the loss of either SAGA or Bdf2 suppresses the sensitivity of TORC2 mutant cells to a variety of stresses, including DNA replication, DNA damage, temperature and nutrient stresses. We suggest a role of TORC2 in transcriptional regulation that is critical for gene silencing and gene induction in response to stress and involves the binding of Gcn5 to the chromatin.

## Author summary

A fundamental characteristic of signaling pathways is their role in the regulation of gene expression. Target of rapamycin (TOR), an atypical protein kinase, is a component of two highly conserved TOR complexes: TORC1 and TORC2. These complexes affect nuclear functions through their activity in the cytoplasm as well as directly, through nuclear TOR pools. We previously showed that in fission yeast, TORC2 and its downstream substrate Gad8 associate with the chromatin fraction. We have also demonstrated that TORC2-Gad8 is required for gene silencing and transcriptional induction in response to stresses. Here, we demonstrate that Gad8 physically associates with proteins of the transcriptional machinery. We found that the loss of two positive transcriptional regulators, the SAGA complex and the bromodomain protein Bdf2, restored gene silencing as well as stress-induced transcriptional induction in TORC2 mutant cells. Our data suggests that TORC2-Gad8 contributes to gene regulation through a mechanism involving the inhibition of the accumulation of Gcn5 at the chromatin. These findings suggest a novel and possibly conserved functional relationship between TORC2, Gcn5 and Bdf2. These proteins and protein complexes are key targets in cancer therapies, thus, deciphering their functional relationship may be valuable for combination therapy in personalized medicine.

## Introduction

Cells display high plasticity of gene expression and chromatin reorganization, which is crucial for responding to stresses [1]. This flexible response requires the cooperation of signal transduction pathways with proteins dedicated to the regulation of chromatin modifications and transcription. Target of rapamycin (TOR) is an atypical serine/threonine protein kinase that acts as the catalytic subunit of the highly conserved complexes: TOR complex 1 (TORC1) and TOR complex 2 (TORC2). Both TOR complexes coordinate cell growth and proliferation with nutritional and stress signals through a wide range of cellular mechanisms [2,3]. Recently, attention has been paid to the roles of TORC1 and TORC2 signaling in the regulation of transcription and the epigenome [4,5].

In fission yeast, *Schizosaccharomyces pombe*, TORC2 contains Tor1 (mTOR in human cells) as its catalytic subunit, together with two essential auxiliary subunits: Ste20 (Rictor in human cells) and Sin1 (mSin1 in human cells) [6,7]. *S*. *pombe* TORC2 is not essential under normal growth conditions but is required for starvation-induced cellular responses, including sexual development, and for cell survival under a variety of stress conditions, including low-glucose, extreme temperatures, osmotic, oxidative, DNA damage or DNA replication stress conditions [8–15]. The roles of *S*. *pombe* TORC2 in nutrient and stress signaling are mediated by its downstream kinase, Gad8 (AKT or SGK1 in human cells) [10,16,17]. Interestingly, unlike stress-responsive pathways, such as the MAP kinase Sty1-dependent pathway (an orthologue of the mammalian p38 MAPK [18]), the TORC2-Gad8 signaling pathway is not activated in response to stress. In contrast, in response to glucose withdrawal or exposure to high osmolarity, the activity of TORC2 or Gad8 is shut down [19] or transiently lost [20,21]. Whereas the Sty1-dependent pathway is known to upregulate stress-responsive gene expression via the Atf1 transcription factor and its partner Pcr1 [18], the molecular mechanism underlying the role of TORC2-Gad8 in transcriptional upregulation in response to stress conditions is far less well understood.

Under normal growth conditions, genome-wide transcriptional profiling of cells lacking the catalytic subunit of TORC2, Tor1 or Gad8, show abnormal upregulation of genes located in subtelomeric regions [22]. Subtelomeric regions are adjacent to constitutive heterochromatic telomeric repeats and contain dimethylated heterochromatic histone H3-lysine-9 (H3K9Me2); this modification gradually declines as the heterochromatin spreads away from the telomeres [23]. The loss of gene silencing at subtelomeric regions in TORC2-Gad8 mutant cells is accompanied by a loss of H3K9Me2 and is suppressed by the loss of Paf1, a subunit of the conserved RNA polymerase associated factor1 complex (Paf1C) [22]. Paf1C is a multifunctional regulator of gene expression, which accompanies RNA polymerase II (Pol II) [24] and antagonizes heterochromatin spreading in *S*. *pombe* [25–27]. Further support for a functional link between TORC2 and Paf1C has been provided by recent studies suggesting that Paf1C is negatively regulated by TORC2, thereby regulating the subtelomeric heterochromatin during quiescence [28].

While TORC2-Gad8 is required for gene silencing, it is also required for gene induction in response to DNA replication stress [11,29]. In *S*. *pombe*, the genes expressed at the G1/S transition are induced by DNA replication stress, and their regulation depends on the *Mlu*I cell-cycle box binding factor (MBF) complex, which is functionally analogous to the mammalian E2F complex [23,30–32]. Cells with *tor1*^+^ or *gad8*^+^ disruption are defective in the upregulation of MBF-regulated gene transcription in response to the DNA replication stress induced by hydroxyurea (HU). This defect is accompanied by decreased binding of the MBF transcription complex to its cognate promoters [29]. Our previous studies have not indicated a link between the effect of TORC2 on MBF-regulated gene transcription and two known transcriptional co-repressors of the MBF complex, Yox1 and Nrm1 [29]. Thus, additional defects apart from the downregulation of MBF recruitment are likely to be involved in the role of TORC2-Gad8 in the induction of MBF-regulated genes.

Here, we show that Gad8 associates with proteins and protein complexes that are closely associated with the transcription machinery, including Paf1C, the co-transcriptional activator SAGA and the bromodomain and extra-terminal motif (BET) protein Bdf2. We demonstrate that Gcn5, the histone acetyl transferase (HAT) subunit of SAGA, accumulates at the chromatin in the absence of TORC2, both at subtelomeric genes and at non-induced MBF-promoters. Removal of Gcn5 by disruption of *gcn5*^+^ rescues the silencing defects in TORC2 mutant cells as well as the lack of MBF gene induction. Disruption of *bdf2*^+^ in TORC2 mutant cells results in reduced levels of Gcn5 at the chromatin, and also rescues the silencing defect and lack of MBF gene induction in these cells. Loss of SAGA subunits (Gcn5 or Ubp8) or Bdf2 suppresses the stress hypersensitivity of TORC2 mutant cells under replication stress, consistent with the suppression of MBF-dependent transcription, but also the hypersensitivity of TORC2 mutant cells under DNA damage, extreme temperatures or low-glucose conditions. Thus, although SAGA and Bdf2 play positive roles in gene transcription in wild type cells, their presence is detrimental to gene upregulation and cell survival in TORC2 mutant cells. We suggest that TORC2-Gad8 has a role in the negative regulation of Gcn5 chromatin loading; this role contributes to gene silencing and becomes critical under stress conditions.

## Results

### Gad8 physically interacts with proteins associated with transcriptionally active euchromatin

**Fig 1:**
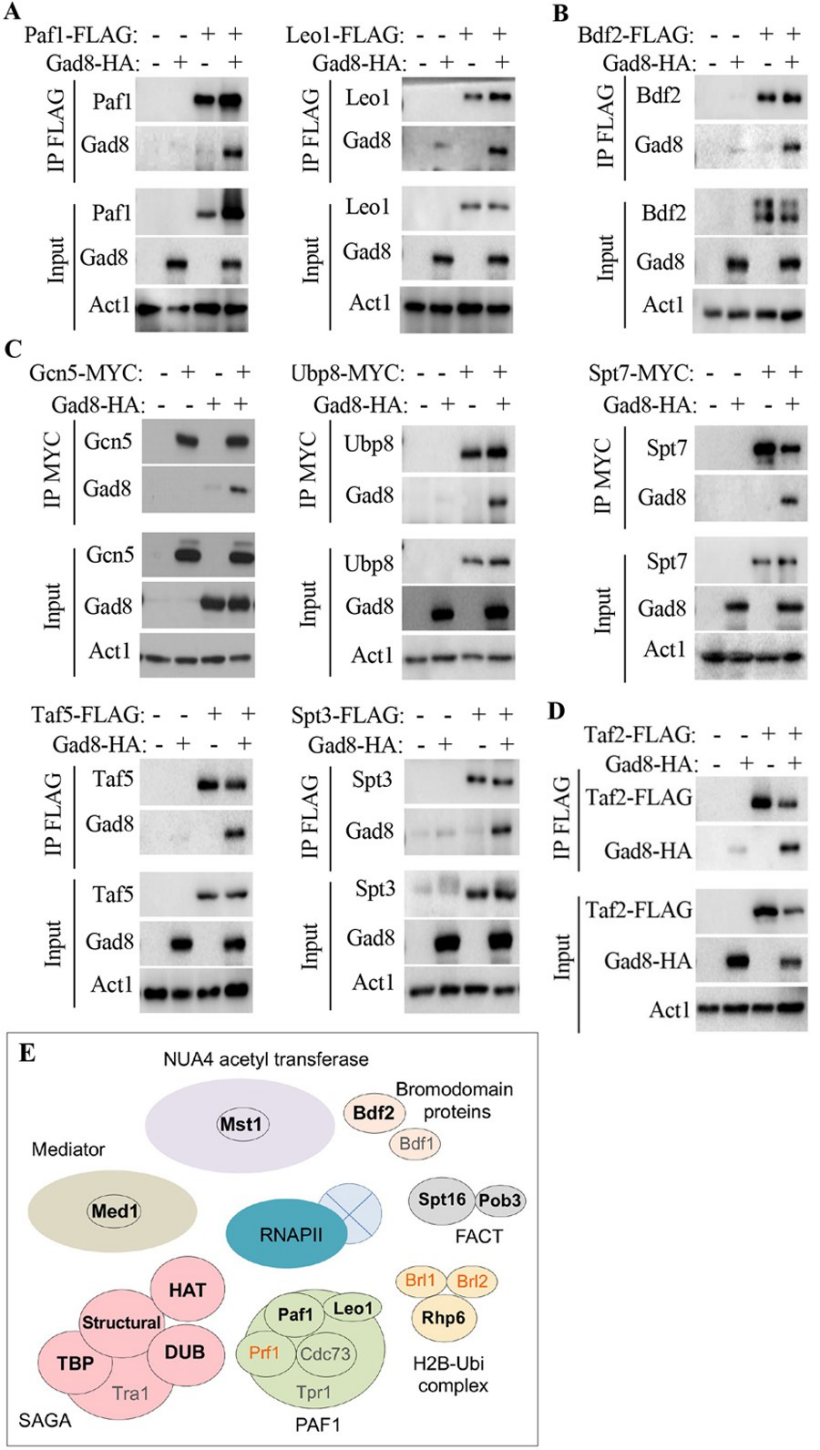
Gad8 associates with transcription and chromatin-modification factors. Gad8 associates with subunits of Paf1C (**A**) Bdf2 (**B**) subunits of SAGA (**C**) and with Taf2, a subunit of TFIID (**D**). Protein extracts from wild type cells expressing the indicated chromosomally tagged proteins were immunoprecipitated (IP) with anti-MYC or anti-FLAG antibody. Western blots were performed using either anti-HA, anti-FLAG or anti-MYC. The expression levels of the indicated proteins before IP is shown (Input). Act1 was used as a loading control. (**E**) A schematic presentation showing proteins that physically interact with Gad8. Bold letters: positive interactions, grey letters: failure to detect interaction and orrange letters: failure to express the tagged protein. Notably, all proteins that interacts with Gad8 are closely associated with RNA Pol II. The light blue circle represents a nucleosome.

We previously showed that *S*. *pombe* cells with disruption of either TORC2 or Gad8, the downstream kinase activated by TORC2, show strong silencing defects at the mating-type locus and at subtelomeric regions [22]. These silencing defects are suppressed by the loss of Paf1 or Leo1, two subunits of Paf1C [22,28]. In another study, we demonstrated that Tor1 and Gad8 are found in the nucleus, in association with the chromatin fraction [29]. Thus, we considered the possibility that Gad8 might physically interact with Paf1C. We used co-immunoprecipitation (co-IP) assays, together with tagged proteins expressed from their endogenous chromosomal locations. We readily detected physical interactions between Gad8 and Paf1 or between Gad8 and Leo1 (Fig 1A). In *S*. *pombe*, Paf1C is minimally composed of four subunits: Paf1, Leo1, Cdc73 and Tpr1 [33]. No interaction was detected between Gad8 and Cdc73 or Tpr1 (S1A Fig). Leo1 and Paf1 have been suggested to form a heterodimer that acts independently of Paf1C with respect to the regulation of heterochromatin propagation [26]. Further studies are required to determine whether Gad8 interacts with Leo1-Paf1 as part of a complete Paf1C, or only with the Leo1-Paf1 sub-complex.

Epe1 is an anti-silencer protein that accumulates at euchromatin-heterochromatin boundaries [34–37] and cooperates with the Paf1-Leo1 heterodimer in regulating heterochromatin spreading [26,27]. Epe1 also recruits the BET bromodomain protein Bdf2 to heterochromatic borders [38]. We previously found that loss of Epe1 partially suppresses silencing defects in TORC2-Gad8 mutant cells [22]. We did not detect a reproducible physical interaction between Gad8 and Epe1 (S1B Fig); however, we readily detected physical interactions between Gad8 and Bdf2 (Fig 1B). *S*. *pombe* expresses two BET bromodomain proteins: Bdf1 and Bdf2, which are involved in efficient acetylation of histone H4 [39] and also act independently: Bdf2 is important for heterochromatin-euchromatin boundary functions [38], whereas Bdf1 acts as part of the SWR1-C chromatin-remodeling complex [40–42]. In co-IP analyses, we did not detect physical interactions between Gad8 and Bdf1 (S1C Fig). However, Gad8 was previously identified as a protein enriched in native chromatin immunoprecipitations that used either Bdf1-Flag or Bdf2-Flag (Supplementary Table S3 in [43]). Thus, Gad8 is likely to interact with both Bdf1 and Bdf2, although the interaction with Bdf1 may be less stable.

Epe1 also recruits the SAGA complex [44]. This multi-protein transcriptional activation complex contains four modules, including a histone acetyl transferase (HAT) and a de-ubiquitinase (DUB) module [45,46]. We examined the physical interactions between Gad8 and representatives of each of the four modules constituting SAGA. We found that Gad8 interacts with Gcn5, the catalytic subunit of the HAT module; Ubp8, the catalytic subunit of the DUB module; Spt7 and Taf5, the subunits of the core structural module; and Spt3, a subunit of the TBP module (Fig 1C and schematic representation in Fig 1E). Gad8 has been found to interact with and to phosphorylate Taf12, a subunit shared by SAGA and the general transcriptional factor complex TFIID [15]. Gad8 phosphorylates Taf12 specifically under nitrogen stress conditions, thereby contributing to the regulation of gene expression during sexual development [15]. Here, we found that Taf2, another subunit of TFIID [47], also interacts with Gad8 (Fig 1D). Thus, our findings further support interactions between Gad8 and the SAGA or TFIID complexes.

The above results led us to test additional interactions between Gad8 and proteins that interact with Pol II, Paf1C or SAGA. We chose representative subunits of Mediator, the essential Mst1 acetyltransferase complex (equivalent to Nua4 in *S*. *cerevisiae*, [48]) and the H2B ubiquitin ligase complex (HULC), which is recruited by Paf1C [33,49]. We found that Gad8 co-immunoprecipitated with Med1, a subunit of Mediator [50]; Mst1 [48]; and Rhp6, the E2 ubiquitin conjugating enzyme of HULC [51] (S2A–S2C Fig). We also examined physical interactions with the E3 ubiquitin ligases of HULC, Brl1 or Brl2. However, the tagged versions of Brl1 and Brl2 were unstable, and therefore we were unable to determine whether such physical interactions exist. The H2B ubiquitylation modifications cooperate with the histone remodeling FACT complex [52] and promote Set1/COMPASS dependent H3K4 methylation [53]. We found that two subunits of the FACT complex, Spt16 and Pob3 [54], interact with Gad8 (S2D Fig), but we detected no interaction with Set1 (S1D Fig).

Taken together, our data demonstrate the association of Gad8 with a subset of positive regulators of transcription initiation and/or elongation, which are closely associated with Pol II (Fig 1E). Using Western blot analyses, we examined whether proteins that interact with Gad8, or their associated partners, show differences in mobility shift in wild type versus Δ*tor1* mutant cells. These could indicate TORC2-dependent phosphorylation. However, none of the examined proteins showed differences in mobility shifts that are dependent on Tor1 (S3 Fig). Thus, although our findings support the presence of Gad8 in the chromatin fraction [29], it remains to be determined whether Gad8 binds the above transcription regulators directly or indirectly.

### Mutations in Paf1C, Mediator, SAGA, Bdf1 or Bdf2 suppress gene de-silencing of subtelomeric genes in TORC2 mutant cells

**Fig 2:**
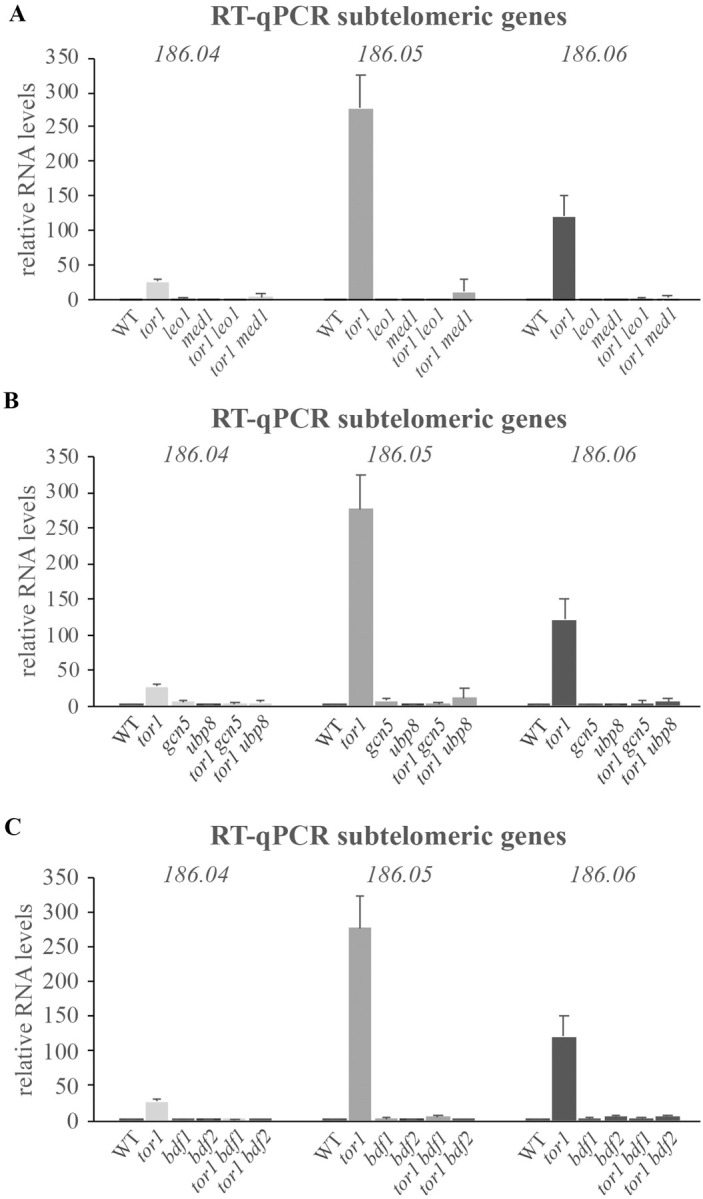
Suppression of the silencing defect in TORC2 mutant cells. Loss of Leo1 or Med1 (**A**), Gcn5 or Ubp8 (**B**) and Bdf1 or Bdf2 (**C**) suppresses the silencing defect of Δ*tor1* cells at subtelomeric genes. Expression levels of 3 subtelomeric genes, *spac186*.*04*, *spac186*.*05* and *spac186*.*06* were determined by RT-qPCR in wild type (WT) and the indicated deletion mutant cells. The level of *act1*^*+*^ mRNA was used as a reference. Each value is the mean of at least three independent assays and the error bars indicate standard deviation.

Because Δ*paf1* or Δ*leo1* have been found to restore gene silencing in TORC2 mutant cells [22,28], we examined whether the deletion of other non-essential Gad8 interactors might also show a similar effect. We examined three subtelomeric genes, *spac186*.*04*, *spac186*.*05* and *spac186*.*06*, showing transcriptional upregulation in Δ*tor1* cells. These genes were previously used to demonstrate that Δ*paf1* restores gene silencing in Δ*tor1* cells [22]. The three subtelomeric genes are located close to the telomeric repeats of the right arm of chromosome I [23]. In Δ*tor1* cells we detected ~30, ~280 and ~120 -fold induction of the transcription of the subtelomeric genes *spac186*.*04*^*+*^, *spac186*.*05*^*+*^ and *spac186*.*06*^*+*^, respectively, compared to wild type cells (Fig 2). As expected, the disruption of *leo1*^+^, encoding the protein partner of Paf1, fully restored gene silencing in Δ*tor1* cells (Fig 2A). Deletion of *med1*^+^ (Fig 2A), *gcn5*^+^ or *ubp8*^+^ (Fig 2B), *bdf1*^+^ or *bdf2*^+^ (Fig 2C) also fully restored gene silencing in Δ*tor1* cells. Thus, single deletion mutations affecting the Paf1C, Mediator or SAGA complexes, or loss of the bromodomain proteins Bdf1 or Bdf2 is sufficient to restore silencing in Δ*tor1* cells at the subtelomeric region.

### Mutations in SAGA or Bdf2 suppress the lack of induction of MBF-regulated genes and the sensitivity of TORC2 mutant cells to replication stress

**Fig 3:**
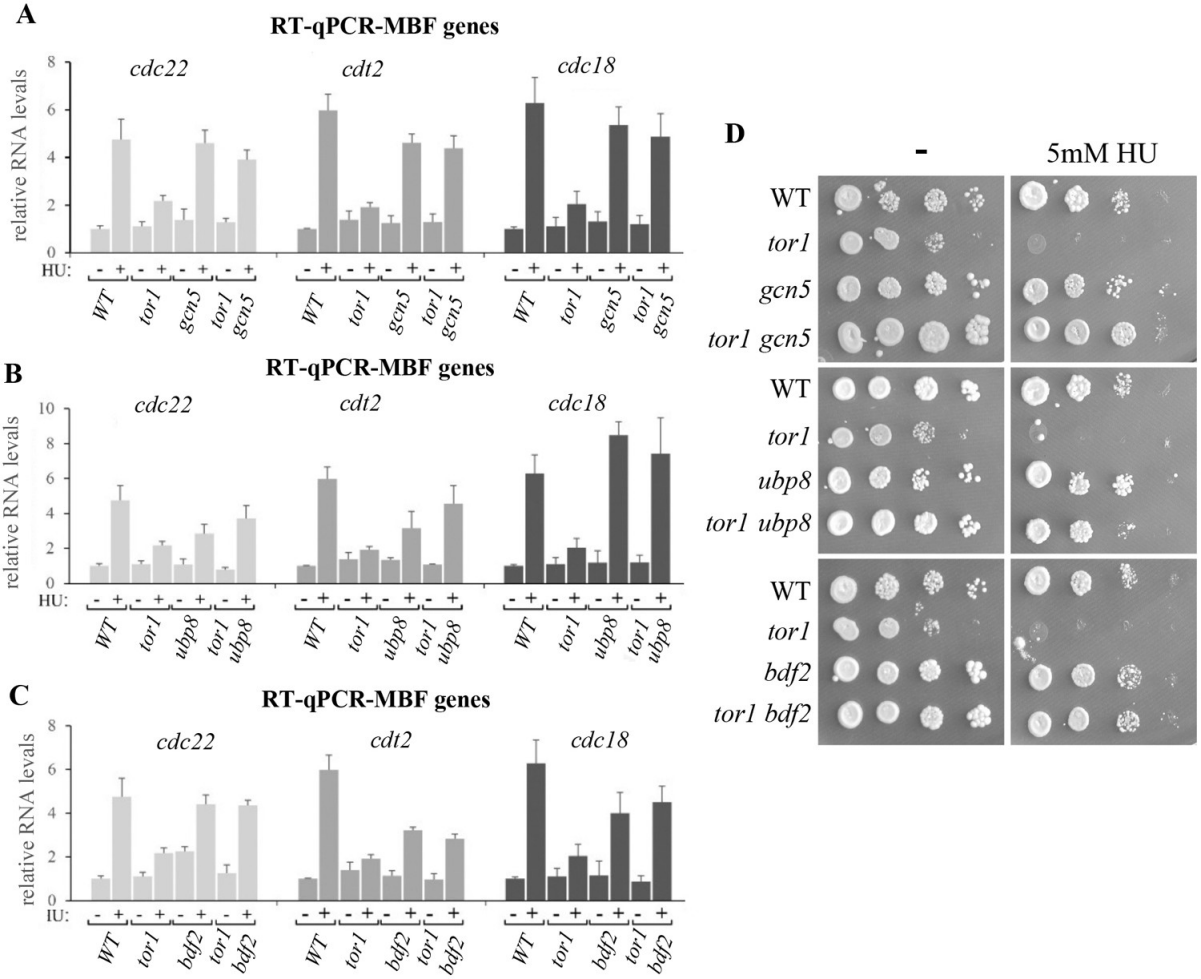
Loss of SAGA or Bdf2 suppress the lack of MBF-regulated gene induction and sensitivity to DNA replication stress in TORC2 mutant cells. (**A-C**) Quantification of transcript levels of *cdc22*^*+*^, *cdt2*^*+*^ and *cdc18*^*+*^ in wild type (WT) and the indicated deletion mutant cells. Total RNA was prepared from untreated cells (-) or cells treated with 12 mM HU for 3 hours (+). The level of *act1*^*+*^ mRNA was used as a reference. Each value is the mean of at least three independent assays, and the error bars indicate standard deviation. (**D**) Serial dilution of exponentially growing wild type and deletion mutant cells were spotted onto minimal medium, with or without 5 mM hydroxyurea (HU).

We previously used HU to induce DNA replication stress and found that TORC2 mutant cells are defective in the induction of several well-established DNA replication stress genes, known in *S*. *pombe* as MBF-regulated genes. These include *cdt2*^*+*^, encoding the adaptor subunit of an E3 ubiquitin ligase, *cdc18*^*+*^, encoding a minichromosome maintenance complex loader and *cdc22*^*+*^, encoding the large subunit of ribonucleotide reductase [29]. Here, we examined whether mutations that restore gene silencing in Δ*tor1* cells might also restore gene induction in response to HU treatment. We found that Δ*gcn5*, Δ*ubp8* or Δ*bdf2* restored MBF-regulated gene induction in Δ*tor1* cells (Fig 3A–3C). In contrast, Δ*paf1*, Δ*leo1* or Δ*med1* were defective or partially defective in the induction of MBF-regulated genes, and did not suppress or only partially suppressed the lack of MBF-regulated gene induction (S4A–S4C Fig). Disruption of *bdf1*^+^ led to abnormally high MBF-regulated gene expression under non-induced conditions, but nevertheless did not suppress gene induction in Δ*tor1* cells (S4D Fig). We reasoned that since either Δ*gcn5*, Δ*ubp8* or Δ*bdf2* rescues the defect in MBF-regulated gene induction, these mutations may also suppress the sensitivity of Δ*tor1* cells to replication stress. Indeed, serial dilution assays demonstrate that Δ*gcn5*, Δ*ubp8* or Δ*bdf2* suppresses the severe sensitivity of Δ*tor1* cells to 5 mM HU (Fig 3D). As previously shown [32], Δ*gcn5* cells are sensitive to HU concentrations higher than 5 mM (S5 Fig). Consistently Δ*gcn5* could not suppress the sensitivity of Δ*tor1* to 6 or 7 mM HU (S5 Fig).

Taken together, our findings indicate that the loss of Gcn5, Ubp8 or Bdf2 in Δ*tor1* cells restored MBF-regulated transcriptional induction and consistently rescued the sensitivity to DNA replication stress in these cells.

### Cells lacking TORC2 accumulate Gcn5 at subtelomeric genes

**Fig 4:**
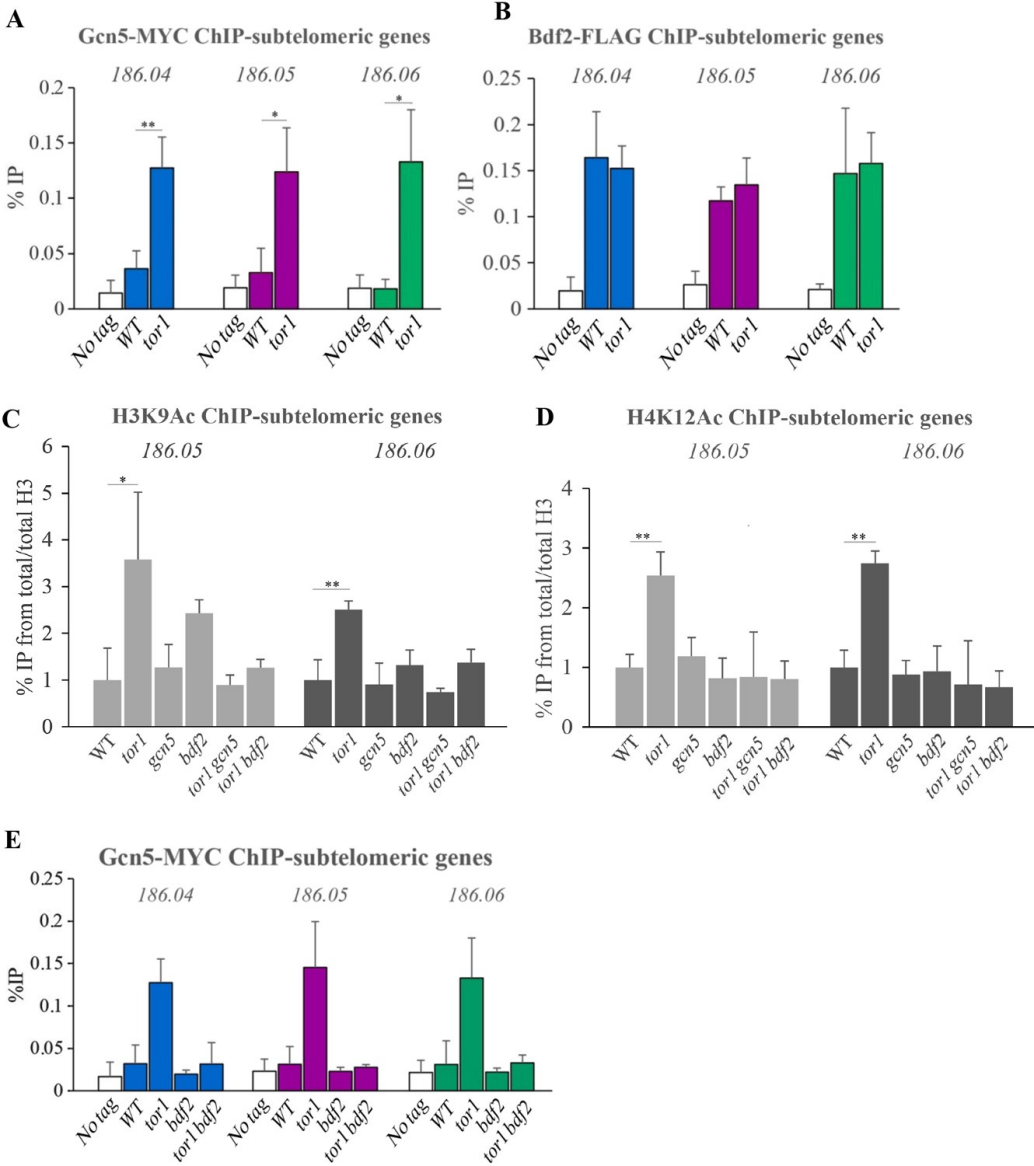
The aberrant accumulation of Gcn5 and transcription-associated chromatin modifications at subtelomeric genes in TORC2 mutant cells is suppressed by loss of Gcn5 or Bdf2. (**A**) Loading of Gcn5 at the subtelomeric genes, *spac186*.*04*^*+*^, *spac186*.*05*^*+*^ and *spac186*.*06*^*+*^ was measured by chromatin immunoprecipitation (ChIP) analysis of chromatin extracts isolated from untagged cells (No tag), cells carrying Gcn5-MYC (WT) or *Δtor1* cells carrying Gcn5-MYC (*tor1*). The level of binding is quantified on anti-MYC immunoprecipitated DNA by quantitative PCR (ChIP-qPCR). (**B**) Loading of Bdf2-FLAG at subtelomeric genes was measured as described for Gcn5-MYC in A. (**C-D**) Levels of H3K9Ac (C) and H4K12Ac (D) were measured by ChIP-qPCR in wild type and deletion mutant cells. (E) Loading of Gcn5-MYC in Δ*bdf2* mutant cells was measured as described in A. Each value is the mean of at least three independent assays, and the error bars indicate standard deviation and significant differences were determined by the Students t-test (*P<0.05, **P<0.01).

Because only mutations in SAGA or Bdf2 restored gene silencing as well as MBF-regulated gene induction, we further concentrated on the regulation of Gcn5 and Bdf2. We hypothesized that TORC2 may affect the chromatin binding or activity of Gcn5 and/or Bdf2. To test this hypothesis, we used chromatin immunoprecipitation analyses followed by quantitative PCR to examine the chromatin binding of Gcn5-MYC or Bdf2-FLAG proteins expressed from their endogenous chromosomal loci. We detected a markedly higher level of Gcn5 binding at subtelomeric genes in Δ*tor1* cells, compared with wild type cells (Fig 4A). In contrast, we found no difference in Bdf2 binding between Δ*tor1* cells and wild type cells at subtelomeric genes (Fig 4B). Gcn5 acetylates multiple histone H3 lysine residues, including lysine 9 (forming H3K9Ac [32,55]). Consistently, we detected higher levels of H3K9Ac at subtelomeric genes in Δ*tor1* cells compared to wild type cells, and this defect was suppressed by either Δ*gcn5* or Δ*bdf2* (Fig 4C). We also examined acetylated histone H4-lysine-12 (H4K12Ac), a histone modification that is catalyzed by Mst1 and is associated with active euchromatin [48]. The H4K12Ac modification is elevated in Δ*tor1* cells, but suppressed by Δ*gcn5* or Δ*bdf2* (Fig 4D). The Δ*bdf2* mutation also suppresses the elevated levels of Gcn5 at the subtelomeric chromatin in Δ*tor1* cells (Fig 4E). Thus, the loss of Bdf2 may lead to the dissociation of Gcn5 from the chromatin. Alternatively, Δ*bdf2* restores gene silencing in Δ*tor1* cells via a Gcn5-independent mechanism, which consequently results in excluding Gcn5 from the chromatin. In both scenarios, however, there is a correlation between the presence of high levels of Gcn5 binding, elevated H3K9Ac and loss of gene silencing at subtelomeric genes.

### Cells lacking TORC2 accumulate Gcn5, but not H3K9Ac, at non-induced MBF promoters

**Fig 5:**
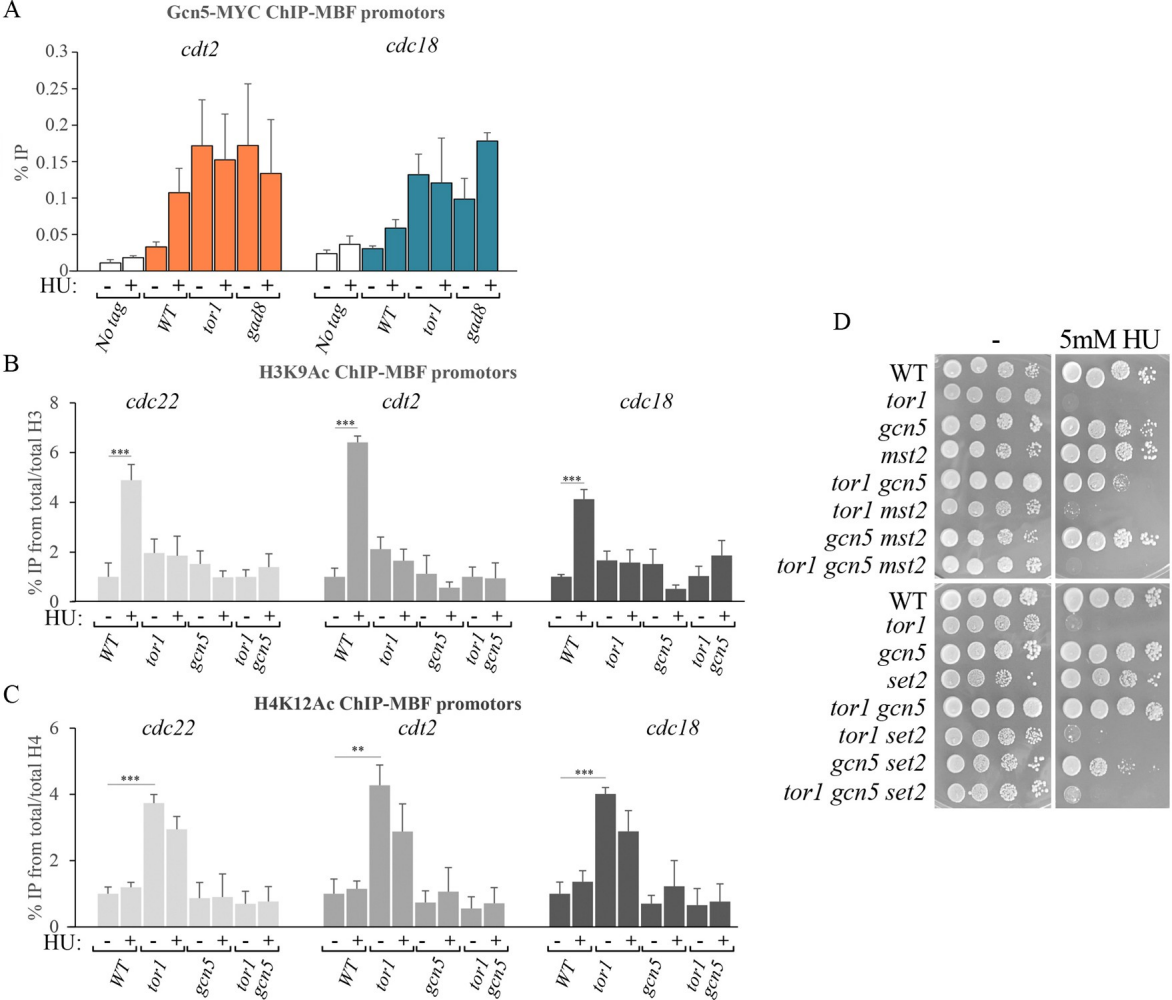
High levels of Gcn5 binding and abnormal transcription-associated chromatin modifications at MBF-regulated promoters in TORC2 mutant cells. (**A**) Loading of Gcn5 at the promoters of *cdt2*^+^ and *cdc18*^+^ was measured by chromatin immunoprecipitation (ChIP) analysis of chromatin extracts isolated from untagged cells (No tag), or wild type cells carrying Gcn5-MYC (WT), or the indicated mutant cells carrying Gcn5-MYC. Cells were untreated (-) or treated with 12 mM HU for 3 hours (+). The level of binding is quantified on anti-MYC immunoprecipitated DNA by quantitative PCR. (B and C) Levels of H3K9Ac (B) and H4K12Ac (C) measured by ChIP-qPCR at MBF promoters in wild type and the indicated deletion mutant cells. Each value is the mean of at least three independent assays, and the error bars indicate standard deviation and significant differences were determined by the Students t-test (*P<0.05, **P<0.01, ***P<0.001). (D) Serial dilution of exponentially growing wild type and deletion mutant cells were spotted onto minimal medium, with or without 5 mM hydroxyurea (HU).

We next tested whether TORC2 also affects the binding of Gcn5 at MBF promoters. Previous studies have demonstrated that Gcn5 is recruited to MBF promoters in response to HU treatment, whereas the level of binding of other histone acetyltransferases, such as Mst1 or Mst2, does not change in response to HU [32]. We successfully reproduced the increased binding of Gcn5 to MBF promoters in response to HU treatment, specifically at *cdc22*^+^ and *cdc18*^+^ (Fig 5A). Unexpectedly, although there is no increase in MBF-dependent transcription, we detected an increase in Gcn5 binding at the promoters of *cdc22*^*+*^ or *cdc18*^*+*^ in Δ*tor1* or Δ*gad8* cells under normal or replication stress conditions (Fig 5A). Thus, the TORC2-Gad8 inhibits the accumulation of Gcn5 at MBF promoters under non-induced conditions, similar to its effect at subtelomeric genes.

The H3K9Ac modification is induced in wild type cells in response to HU ([32], Fig 5B). In contrast, in Δ*tor1* cells, despite the high levels of Gcn5 binding, no increase, or only a small increase, is observed in H3K9Ac under non-induced conditions, and no induction of H3K9Ac is observed in response to HU (Fig 5B). Thus, in the context of the MBF promoters, the activity of Gcn5 in Δ*tor1* cells may be compromised. Alternatively, Gcn5 is active at MBF promoters, but the H3K9Ac fails to accumulate, for example due to higher activity of histone de-acetylation. Other, non-histone substrates of Gcn5 [56] may also be affected by the accumulation of Gcn5 at the chromatin in TORC2-Gad8 mutant cells. Examination of the H4K12Ac modification, which is normally associated with transcription, revealed that this modification is significantly elevated at MBF promoters in Δ*tor1* cells, either under non-induced or induced conditions (Fig 5C). The abnormally elevated levels of H4K12Ac were suppressed by Δ*gcn5* (Fig 5C). Since H4K12Ac is induced by Mst1, we examined whether a temperature sensitive mutations in *mst1*, *mst1-L244S*, could suppress HU sensitivity in Δ*tor1* cells. We found that the *mst1-L244S* mutation does not suppress the sensitivity of Δ*tor1* cells to HU (S6 Fig). This finding does not support a scenario in which increased Mst1-dependent activity underlies the defect in MBF-dependent gene induction in Δ*tor1* cells.

Set2 was implicated in MBF-dependent transcription [57], while Mst2 acts redundantly with Gcn5 in promoting H3K14 acetylation [58]. Interestingly, we found that disruption of either *mst2*^+^ or *set2*^+^ reversed the suppression of Δ*tor1* by Δ*gcn5* (Fig 5D). These findings may suggest that removal of non-productive Gcn5 in Δ*tor1* cells allows the binding and function of Set2 and Mst2, which are otherwise not essential for the DNA replication response in wild type cells. Alternatively, in Δ*gcn5* Δ*tor1* double mutant cells, the transcription of MBF-dependent gens is precarious and thus becomes dependent on the activities of Set2 and Mst2, independent of removal of Gcn5 from the chromatin.

### Loss of Bdf2 restores normal Gcn5 binding at MBF promoters

**Fig 6:**
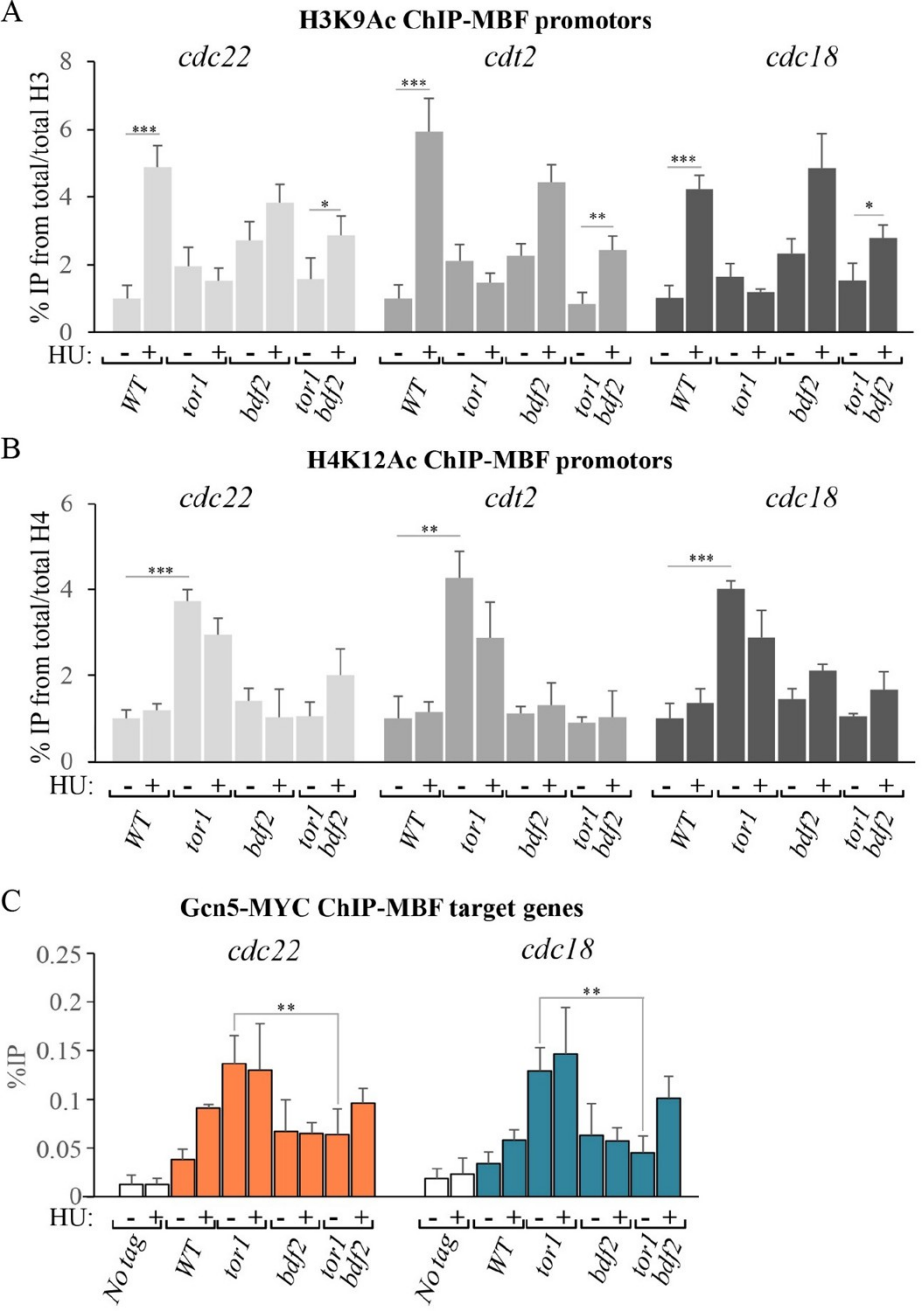
Loss of Bdf2 partially suppresses aberrant histone chromatin modifications and high levels of Gcn5 binding in TORC2 mutant cells at MBF promoters. **(A-B)** Levels of H3K9Ac (A) and H4K12Ac (B) measured by ChIP-qPCR at MBF promoters in wild type and the indicated deletion mutant cells. **(C)** Loading of Gcn5 at the promoters of *cdc22*^+^ and *cdc18*^+^ was measured by chromatin immunoprecipitation (ChIP) analysis of chromatin extracts isolated from untagged cells (No tag), wild type cells carrying Gcn5-MYC (WT), or the indicated mutant cells carrying Gcn5-MYC. Cells were untreated (-) or treated with 12 mM HU for 3 hours (+). The level of binding is quantified on anti-MYC immunoprecipitated DNA by quantitative PCR. Each value is the mean of at least three independent assays, and the error bars indicate standard deviation and significant differences were determined by the Students t-test (**P<0.01, ***P<0.001).

Our data indicate that Δ*bdf2* suppresses the lack of MBF gene induction in Δ*tor1* cells, similar to Δ*gcn5* (Fig 3C). Thus, we examined which chromatin defects in Δ*tor1* cells are suppressed by Δ*bdf2*. The Δ*bdf2* mutation partially suppressed the lack of induction of H3K9Ac (Fig 6A) and the high levels of H4K12Ac (Fig 6B). Bdf2 itself is bound to MBF promoters in wild type cells, but its levels are not increased in response to HU, and are moderately reduced in response to HU in Δ*tor1* cells (S7 Fig). Since Δ*bdf2* suppresses MBF-dependent transcription in Δ*tor1* cells, the reduction in the levels of Bdf2 at MBF promoters in Δ*tor1* cells in the presence of HU does not seem relevant to its suppression activity. Significantly, Δ*bdf2* restores low levels of Gcn5 binding at MBF promoters in Δ*tor1* cells under normal growth conditions and also restores the normal pattern of an increased level of Gcn5 in response to HU (Fig 6C).

### Loss of Gcn5 or Bdf2 suppresses the stress hypersensitivity and sterility in TORC2 mutant cells

**Fig 7:**
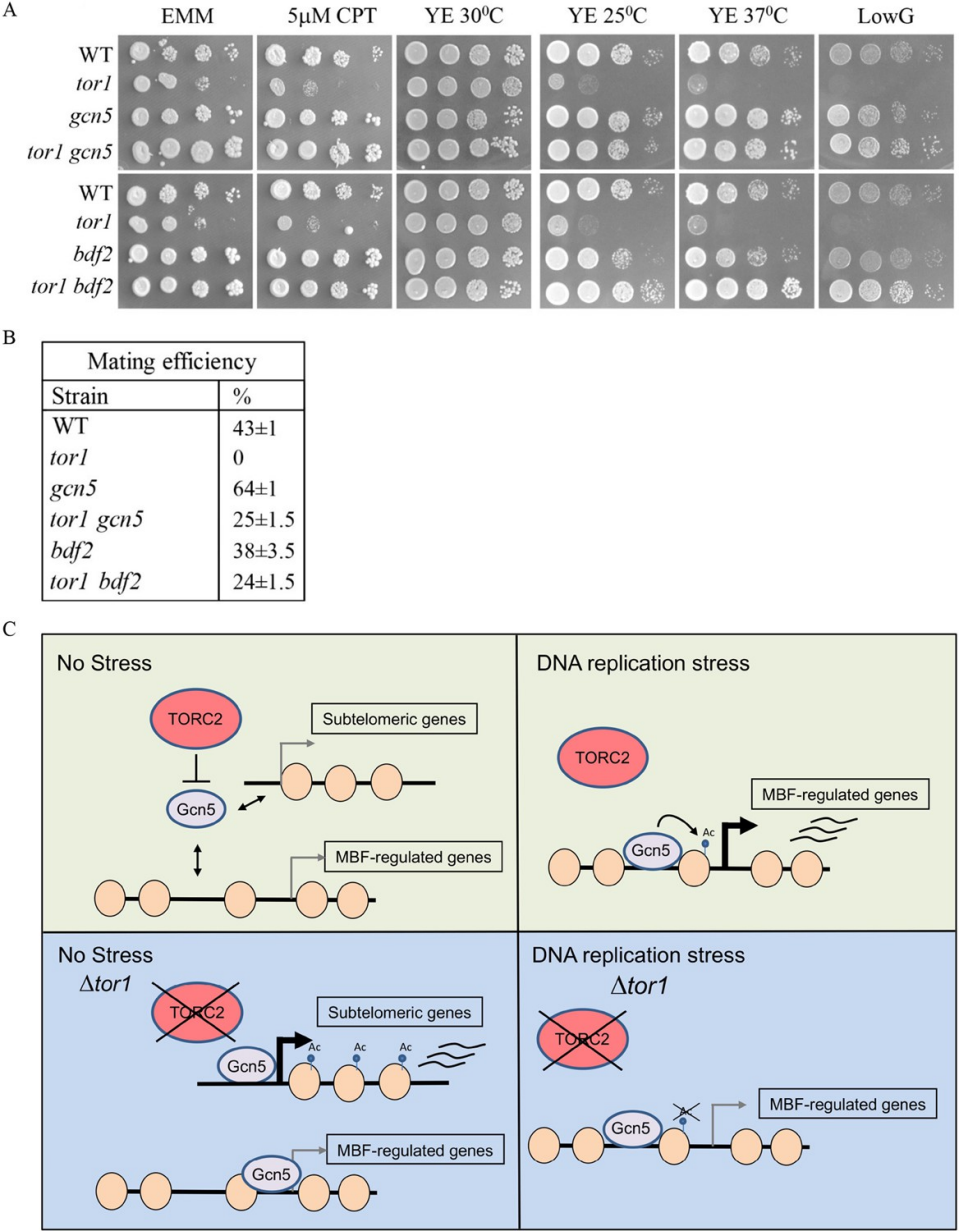
Loss of Gcn5 or Bdf2 suppresses the hypersensitivity and sterility of TORC2 mutant cells. (**A**) Serial dilution of exponentially growing wild type and deletion mutant cells were spotted onto minimal medium (EMM), with or without camptothecin (CPT) or onto rich medium (YE) at standard temperature (30°C), low temperature (25°C), high temperature (37°C) or YE medium in which glucose was replaced with 2% galactose and 0.1% glucose (LowG). (**B**) Mating efficiency was determined on SPA plates as described in Material and Methods. The results are the mean values of three separate experiments. (**C**) A schematic illustration depicting the effects of TORC2 on Gcn5 loading and gene transcription at subtelomeric and MBF-regulated genes under normal and DNA replication stress.

TORC2 mutant cells are sensitive to diverse stress conditions, including DNA replication stress, DNA damage stress, extreme temperatures and low-glucose conditions. Strikingly, the loss of Gcn5 or Bdf2 suppresses the sensitivity of Δ*tor1* cells not only to DNA replication stress (Fig 3D), but also to DNA damage induced by camptothecin, high or low temperatures (25°C or 37°C), or low glucose conditions (0.1% glucose) (Fig 7A). Δ*gcn5* also suppresses the sensitivity of Δ*gad8* under all these stress conditions (S8 Fig). Somewhat surprisingly, Δ*bdf2* fails to suppress stress sensitivities in Δ*gad8* cells (S8 Fig), suggesting that Gad8 may have additional functions under stress conditions that are independent of Tor1 and that require Bdf2.

TORC2-Gad8 mutant cells fail to enter the sexual development pathway [8–12,14,15], another stress induced program that requires substantial transcriptional reprogramming [59]. Δ*gcn5 or* Δ*bdf2* partially suppresses the sterility of Δ*tor1* cells (Fig 7B), suggesting that similarly to other stress conditions, the presence of Gcn5 or Bdf2 may be deleterious in TORC2 mutant cells with respect to the upregulation of the sexual development transcriptional response. Indeed, it was previously shown that Δ*tor1* or Δ*gad8* mutant cells do not upregulate the transcription of two major key regulators of sexual development, *ste11*^+^ and *mei2*^+^, and these transcriptional defects are suppressed by Δ*gcn5* [15]. It was reported that Δ*gcn5* partially suppresses the sterility of Δ*gad8* but not Δ*tor1* cells [15]. As indicated above, in our experiments, Δ*gcn5* partially suppresses the sterility observed in Δ*tor1* cells (Fig 7B), in line with the induction of *ste11*^+^ and *mei2*^+^ in these genetic backgrounds. Taken together, our findings extend the roles of the SAGA complex downstream of TORC2-Gad8 signaling and suggest a similar involvement of the Bdf2 protein.

## Discussion

TORC2-Gad8 signaling has profound effects on transcriptional regulation under normal and stress conditions [5,11,14,15,22,60], yet little is known about the underlying mechanism. Here, we demonstrate physical interactions between Gad8 and multiple proteins closely associated with the transcription machinery. The proteins associated with Gad8 include components of TFIID, Mediator, SAGA, Paf1C, HULC and FACT. These findings further support the presence of a subpopulation of TORC2-Gad8 in the nucleus [14,29]. Although the novel physical interactions that we identified for Gad8 may either reflect direct or indirect physical interactions, they suggest a physical proximity of TORC2-Gad8 and the transcriptional machinery. Remarkably, we found that loss of two positive transcriptional regulators, either Gcn5 or Bdf2, suppresses aberrant gene transcription and stress hypersensitivity in TORC2 mutant cells. This is, to our knowledge, the first report of suppressors that rescue the hypersensitivity of TORC2-Gad8 mutant cells to a wide variety of different stress conditions.

### Gene silencing at subtelomeres

Heterochromatin is found in *S*. *pombe* cells at centromeres, telomeres and the mating-type locus [61]. The centromeres and the mating-type locus are constrained within well-defined borders, whereas telomeric heterochromatin gradually dissolves as it spreads away from the telomeric repeats and into the chromosome [61]. Mutations in TORC2-Gad8 lead to shrinkage of the H3K9Me2 heterochromatic marker at subtelomeric region and to de-silencing of the telomere-distal genes [[22] and Fig 2]. TORC2-Gad8 also affects gene silencing of reporter genes at the mating type locus [22]. More recently, Gad8 and Cdt2 have been identified in the same screen as regulators of heterochromatin spreading at the mating type locus [62]. Since TORC2-Gad8 controls the level of Cdt2 [29], an interesting possibility is that TORC2-Gad8 controls the level of Epe1 via Cdt2-dependent degradation [37]. At subtelomeric genes, where no clear heterochromatin-euchromatin borders exist, mutations in Paf1C, SAGA, Bdf1 or Bdf2 restore silencing of TORC2 mutant cells (Fig 2). Therefore, in this chromatin context, TORC2-Gad8 may affect heterochromatin spreading by regulating the transcriptional machinery and its associated factors. This possibility is in accordance with the idea that euchromatic signals repel heterochromatin spreading [63]. Gcn5, but not Bdf2, is depleted from subtelomeric genes in wild type cells (Fig 4A–4B), thus suggesting that the regulation of Gcn5 binding is a critical factor for the regulation of gene silencing at subtelomeres (schematic representation in Fig 7C). What mechanism underlies the accumulation of Gcn5 at subtelomeres in TORC2 mutant cells remains to be examined. A mechanism that involves the regulation of Epe1 cannot be ruled out at this stage, however, Δ*epe1* only partially suppresses the silencing defect in TORC2 mutant cells [22].

### Transcriptional gene induction and survival under stress conditions

We demonstrate that cells lacking TORC2-Gad8 abnormally accumulate Gcn5 at non-induced promoters of DNA replication genes (schematic representation in Fig 7C). Gcn5 is normally recruited to MBF promoters in response to DNA replication stress via Rep2, a subunit of the MBF complex, and this recruitment is abolished by deletion of the repressors *yox1*^+^ or *nrm1*^+^ [32]. Since we have previously shown that there is a reduction in the recruitment of the MBF complex in TORC2 mutant cells, the increase in Gcn5 binding in TORC2 mutant cells is unlikely dependent on Rep2. Furthermore, TORC2-Gad8 does not affect the phosphorylation state of Yox1, which regulates its activity as a repressor and disruption of *yox1*^+^ or *nrm1*^+^ does not suppress the lack of MBF induction in TORC2-Gad8 mutant cells [29]. There are multiple mechanisms involved in SAGA recruitment, including through interactions with the transcription machinery and chromatin markers [64]. Thus, we speculate that Gcn5 is aberrantly recruited or retained at the chromatin in TORC2 mutant cells in a mechanism independent of MBF binding. As it was previously shown that Tor1 phosphorylates the Taf12 subunit of SAGA [15], TORC2 may directly regulate SAGA activity or binding. However, so far we have not detected changes in the mobility shift of SAGA subunits in TORC2 mutant cells (S3 Fig). Therefore, how TORC2 inhibits the binding of Gcn5 to the chromatin remains an open question.

Our studies indicate that in Δ*tor1* cells the binding of Gcn5 to MBF promoters is not correlated with an increase in H3K9 acetylation. This finding suggests that the activity of Gcn5 is compromised at MBF promoters in Δ*tor1* cells (but not at subtelomeres). Indeed, the binding of SAGA at the MBF promoters may be aberrant due to lower levels of the MBF complex, which may not allow for proper H3K9 acetylation. Alternatively, Gcn5 is active in Δ*tor1* cells, but the H3K9Ac modification fails to accumulate at MBF promoters, for example due to elevated levels of histone de-acetylation activity.

What mechanism might explain the suppression observed in TORC2 mutant cells by positive regulator of transcription? One possibility is that removal of non-productive Gcn5, either by deleting of *gcn5*^+^ or, indirectly, by Δ*bdf2*, allows the binding of other chromatin modifiers that can support MBF transcription, such as Set2 and Mst2 (Fig 5D). However, since Δ*gcn5* or Δ*bdf2* suppresses the sensitivity of TORC2 mutant cells under many different stresses, it is more tempting to speculate that a general role of Gcn5 and Bdf2 in regulating transcription is responsible for the suppression activities observed in TORC2 mutant cells. Gcn5 in particular has been implicated in survival under various stress conditions in *S*. *pombe* [65]. Moreover, Gcn5 regulates gene expression in response to stresses [32,66,67] and is recruited to the promoters of stress genes, including those that induce oxidative stress response genes [68], and has been suggested to facilitate progression of Pol II to allow induction of gene expression [68]. Both SAGA and Bdf2 have been strongly implicated in transcription elongation [69]. Thus, it is possible that it is the slowing of transcription elongation that is relevant for the rescue of transcription deregulation and stress sensitivity in TORC2 mutant cells. Transcriptional programs executed after acute stress are subject to rigorous transcriptional checkpoint and quality control mechanisms [1]. Such mechanisms require a fine balance between histone modifications that promote or inhibit Pol II progression. We speculate that such equilibrium is disrupted in TORC2-Gad8 mutant cells. Consequently, Gcn5 and Bdf2, which normally play a positive role in promoting transcription, become deleterious in the background of TORC2-Gad8 mutant cells.

### Implications of TORC2, SAGA and BET protein functions in higher eukaryotes

Mammalian TORC2 (mTORC2), SAGA and the family of BET proteins are strong candidates for anti-cancer therapy. mTORC2 primarily functions as an effector of the insulin/PI3K signaling [3] and consequently is specifically involved in the development of cancer induced by upregulation of insulin/PI3K signaling [70]. mTORC2 governs cancer development via regulation of the activity of downstream kinases, AKT [71], independently of AKT [72] and by affecting metabolic reprograming [73] and cellular migration [74]. Human SAGA and BRD4, the mammalian homologue of *S*. *pombe* Bdf2, have been implicated in the transcriptional regulation of oncoproteins and tumor suppressors [45,75]. In particular, de-regulation of BRD4 has been associated with several cancer types, and BET inhibitors are potential drugs for anti-cancer and immune diseases [75]. Our study suggests that exploring the functional relationships among these cellular components in higher eukaryotes may have implications for developing strategies for anti-cancer treatments. Thus, for example, if loss of SAGA or BRD4 in human cells render cells lacking TORC2 resistant to stress, treating cancer cells that have lost TORC2 signaling with drugs that inhibit SAGA or BET family proteins may result in adverse effects of chemotherapy resistance.

## Materials and methods

### Yeast strains, media and growth assays

*S*. *pombe* strains used in this paper are listed in S1 Table. Yeast cells were cultured in rich YE medium supplemented with adenine and uracil or in Edinburgh minimal medium (EMM, 5 g/liter NH_4_Cl), as described in [76]. EMM was supplemented with amino acids according to the auxotrophic requirements of the strains used. For cell growth assays, logarithmic growing cells were serially diluted and spotted on YE or EMM plates, as indicated. Plates were incubated at 30°C, unless otherwise indicated. Gene deletions and tagging were performed by standard PCR-based methods [77]. Oligonucleotides used for gene deletions and protein tagging are listed in S2 Table.

### Assays for mating efficiency

Cells were grown at 30°C in EMM with adequate amino acid supplements to a density of approximately 5x10^6^ cells/ml. Cells were then spotted on SPA plates [76] and incubated for three days at 25°C. An aliquot was taken for inspection under a light microscope and the numbers of cells, zygotes, and spores were counted. The percentage of mating was calculated by dividing the number of zygotes, asci, and free spores by the number of total cells. One zygote or one ascus was counted as two cells, and one spore was counted as a half cell. In each experiment 500 to 1,000 cells were counted.

### Protein extraction and immunoprecipitation (IP) assays

Cells were grown to mid-logarithmic phase, washed once with water and re-suspended in a lysis buffer (PBS pH7.0, 200mM NaCl, 0.5mM EGTA, 0.5mM EDTA, 0.1% Triton X100, protease inhibitor cocktail and 1mM phenylmethylsulfonyl fluoride). Cells were broken by bead beating with glass beads for 45 minutes at 4°C, centrifuged for five minutes at 1000g and the supernatant was collected. 20μg of total protein extract was resolved by SDS-PAGE using 10–15% acrylamide gels. For immunoprecipitations (IP), 500 μg of proteins were prepared and pre-cleared with a 20μl protein A sepharose and protein G sepharose beads mixture (GE Healthcare). 2μl of MYC (Santa cruz, SC40) or FLAG (Sigma, F1804) antibodies were added to the cleared extract and incubated overnight at 4°C. The beads were washed five times with lysis buffer at NaCl concentration of 220mM. The resulting immunoprecipitates were loaded on SDS-PAGE using 10–15% acrylamide gels. TCA protein extraction was performed as described in Foiani et al. [78].

### Western blotting

Proteins were resolved by SDS-PAGE 10–15% acrylamide gels and transferred to nitrocellulose membranes, blocked with 5% milk in TBST and immunoblotted with anti-HA (Santa cruz, SC7392), anti-Actin (MBP, #08691001), anti-MYC (Santa cruz, SC40) or anti-FLAG (Sigma, F1804) as indicated. Detection was carried out using the ECL SuperSignal detection system (Thermo Scientific).

### Real time quantitative PCR (qRT-PCR)

RNA extractions and qRT-PCR analysis were performed as described in [79]. 50 ml of each strain were grown to an A600 of ≈1 in minimal medium. RNA was prepared using the hot phenol method and treated with RNase free RQ1 DNase I (Promega) to remove DNA prior to reverse transcription. 1μg of RNA was reverse-transcribed with ImProm-II Reverse Transcriptase (Promega), followed by Real-time PCR with Precision Fast qPCR Master mix kit (Primer Design). Reactions were performed in triplicate and run on the Step One Plus Real-Time PCR (Applied Biosystems). Threshold cycle (Ct) values for the cDNA of interest were normalized to the Ct values of *act1*^*+*^ and relative expression levels were quantified using the comparative method and calculated as 2^−ΔΔCt^. The amount of expression was expressed relative to the expression level of wild type cells grown in minimal medium (relative value = 1). Oligonucleotides used for qRT-PCR analyses are listed in S3 Table.

### Chromatin immunoprecipitation (ChIP)

50 ml of each strain were grown to OD600 ≈ 1 in YE. 1.5ml formaldehyde (37% solution) was added for 15 minutes and the formaldehyde was quenched with 2.5ml of 2.5M glycine for five minutes. Cells were harvested, washed once with 15ml cold PBS and broken down for 10 minutes with glass beads in 600 μl lysis buffer (50mM HEPES-KOH pH7.5, 140mM NaCl, 1mM EDTA, 1% triton X100, 0.1% Na-Deoxycholic acid). The supernatant was removed to a new tube (the lysate). The glass beads were washed with 500 μl lysis buffer, centrifuged and the supernatant was added to the lysate. The lysate was sonicated six times for 10 seconds at 80% amplitude with one minute on ice between each time. The sonicated material was centrifuged for 30 minutes at 2500 rpm. The supernatant was used for immunoprecipitations (IP). The sonicated proteins were pre-cleared with a 25μl protein A sepharose and protein G sepharose beads mixture (GE Healthcare) and the appropriate antibodies were added to the cleared extract and incubated overnight at 4°C. Gcn-13MYC, Bdf2-5FLAG, H3K4Me3, H3K9Ac, H4K12Ac, H3 and H4, were immunoprecipitated with 2–5μg of antibody of anti-MYC (Abcam ab32), anti-Flag (Sigma, F3165), anti-H3K4me3 (Abcam, ab8580), anti-H3K9Ac (Abcam ab4441), anti-H4k12Ac (Abcam ab46983), anti-H3 (Abcam, ab1791) and anti-H4 (Abcam ab10158). A total of 10% of the extract was saved as input. The beads after the IP were washed once with lysis buffer, once with lysis buffer with 360 mM NaCl, once with washing buffer (10mM Tris/HCl pH8, 0.25M LiCl, 0.5% NP40, 0.5% Na-Deoxycholic acid, 1mM EDTA) and once with TE (10mM Tris/HCl pH8 and 10mM EDTA). The washed beads and the input were treated with elution buffer (50mM Tris/HCl pH8, 10mM EDTA, 1% SDS) overnight at 65°C. The DNA was precipitated, re-suspended in water and used for PCR real-time analysis. All experiments are plotted as the average of at least three independent biological repeats and each biological repeat is the average of three technical PCR repeats. Oligonucleotides used for ChIP analyses are listed in S4 Table.

## Supporting information

S1 Fig Transcription and chromatin-modification factors that do not associate with Gad8. Gad8 does not associate with members of the Paf1C complex, Tpr1 and Cdc73 (**A**), Epe1 (**B**), Bdf1 (**C**) and Set1 (**D**). Protein extracts from wild type cells expressing the indicated chromosomally tagged proteins were immunoprecipitated (IP) with anti-MYC or anti-FLAG antibody. Western blots were performed using either anti-HA, anti-FLAG or anti-MYC to detect the presence of tagged proteins within the immune complexes. The expression levels of the indicated proteins before IP is shown (Input). Act1 was used as a loading control.
(TIF)

S2 Fig Gad8 associates with transcription and chromatin-modification factors. Gad8 associates with Med1 (**A**), Mst1 (**B**), Rhp6 (**C**) and the subunits of the FACT complex, Spt16 and Pob3 (**D**). Protein extracts from wild type cells expressing the indicated chromosomally tagged proteins were immunoprecipitated (IP) with anti-MYC or anti-FLAG antibody. Western blots were performed using either anti-HA, anti-FLAG or anti-MYC to detect the presence of tagged proteins within the immune complexes. The expression levels of the indicated proteins before IP is shown (Input). Act1 was used as a loading control.
(TIF)

S3 Fig Screen for potential TORC2-dependent phosphorylation substrates. Wild type cells expressing the indicated chromosomally tagged proteins were grown to mid-logarithmic phase. The proteins were extracted with TCA and loaded on Phos-tag gels. Western blot analyses were performed using either anti-MYC or anti-FLAG. Act1 was used as a loading control. We detected mobility shifts for several proteins (Bdf2, Taf5 and Pob3), suggesting that these proteins are subjected for phosphorylation, however, no differences were observed in Δ*tor1* cells.
(TIF)

S4 Fig Mutations in Paf1C, Med1 or Bdf1 do not suppress the lack of MBF-dependent gene induction in response to hydroxyurea. (**A-D**) Expression levels of *cdc22*^*+*^, *cdt2*^*+*^ and *cdc18*^*+*^ in wild type (WT) and indicated deletion mutant cells were determined by RT-qPCR. Total RNA was prepared from untreated cells (-) or cells treated with 12 mM HU for 3 hours (+). The level of *act1*^*+*^ mRNA was used as a reference. Each value is the mean of at least three independent assays, and the error bars indicate standard deviation.
(TIF)

S5 Fig Disruption of *gcn5*^+^ does not rescue the sensitivity of Δ*tor1* cells to high concentrations of hydroxyurea. Serial dilution of exponentially growing wild type, Δ*tor1*, Δ*gcn5* or Δ*tor1* Δ*gcn5* cells were spotted onto minimal medium, with or without 5, 6 or 7 mM hydroxyurea (HU). Δ*gcn5* cells are sensitive to 6 or 7 mM HU. The Δ*gcn5* mutation rescues the sensitivity of Δ*tor1* cells at 5 mM HU, but not at higher HU concentrations.
(TIF)

S6 Fig Mutations in *mst1* do not suppress replication stress sensitivity in TORC2 mutant cells. Serial dilution of exponentially growing wild type, Δ*tor1*, *mst1*^*ts*^ or Δ*tor1 mst1*^*ts*^ cells were spotted onto minimal medium, with or without 5 mM hydroxyurea (HU). The *mst1*^*ts*^ strain is resistant to 5 mM HU, but sensitive to camptothecin (CPT). The *mst1*^*ts*^ mutation does not rescue the sensitivity of Δ*tor1* to either HU or CPT.
(TIF)

S7 Fig Bdf2 is not accumulated at MBF promoters in response to replication stress, while its binding to chromatin in Δ*tor1* cells is moderately compromised. Loading of Bdf2 at the promoters of *cdc22*^+^, *cdt2*^*+*^ and *cdc18*^+^ was measured by chromatin immunoprecipitation (ChIP) analysis of chromatin extracts isolated from untagged cells (No tag), wild type cells carrying Bdf2-FLAG (WT) or Δ*tor1* cells carrying Bdf2-FLAG (*tor1*). Cells were untreated (-) or treated with 12 mM HU for 3 hours (+). The level of binding is quantified on anti-FLAG immunoprecipitated DNA by quantitative PCR.
(TIF)

S8 Fig Loss of Gcn5, but not Bdf2, suppresses the sensitivity of Δ*gad8* mutant cells to various stress conditions. Serial dilution of exponentially growing wild type and deletion mutant cells were spotted onto minimal medium (EMM), with or without hydroxyurea (HU) camptothecin (CPT) or onto rich medium (YE) at standard temperature (30°C), low temperature (25°C), high temperature (37°C) or YE medium in which glucose was replaced with 2% galactose and 0.1% glucose (LowG).
(TIF)

S1 Table Strains used in this study. (DOCX)

S2 Table Oligonucleotides used for gene deletions and protein tagging. (DOCX)

S3 Table Oligonucleotides used for qRT-PCR analyses. (DOCX)

S4 Table Oligonucleotides used for ChIP analyses. (DOCX)
