# Supplementary figures and images for: TOR complex 2 contributes to regulation of gene expression via inhibiting Gcn5 recruitment to subtelomeric and DNA replication stress genes
Source: PLoS Genet. 2022 Feb 14;18(2):e1010061. doi: 10.1371/journal.pgen.1010061 (PMC8880919; doi:10.1371/journal.pgen.1010061)

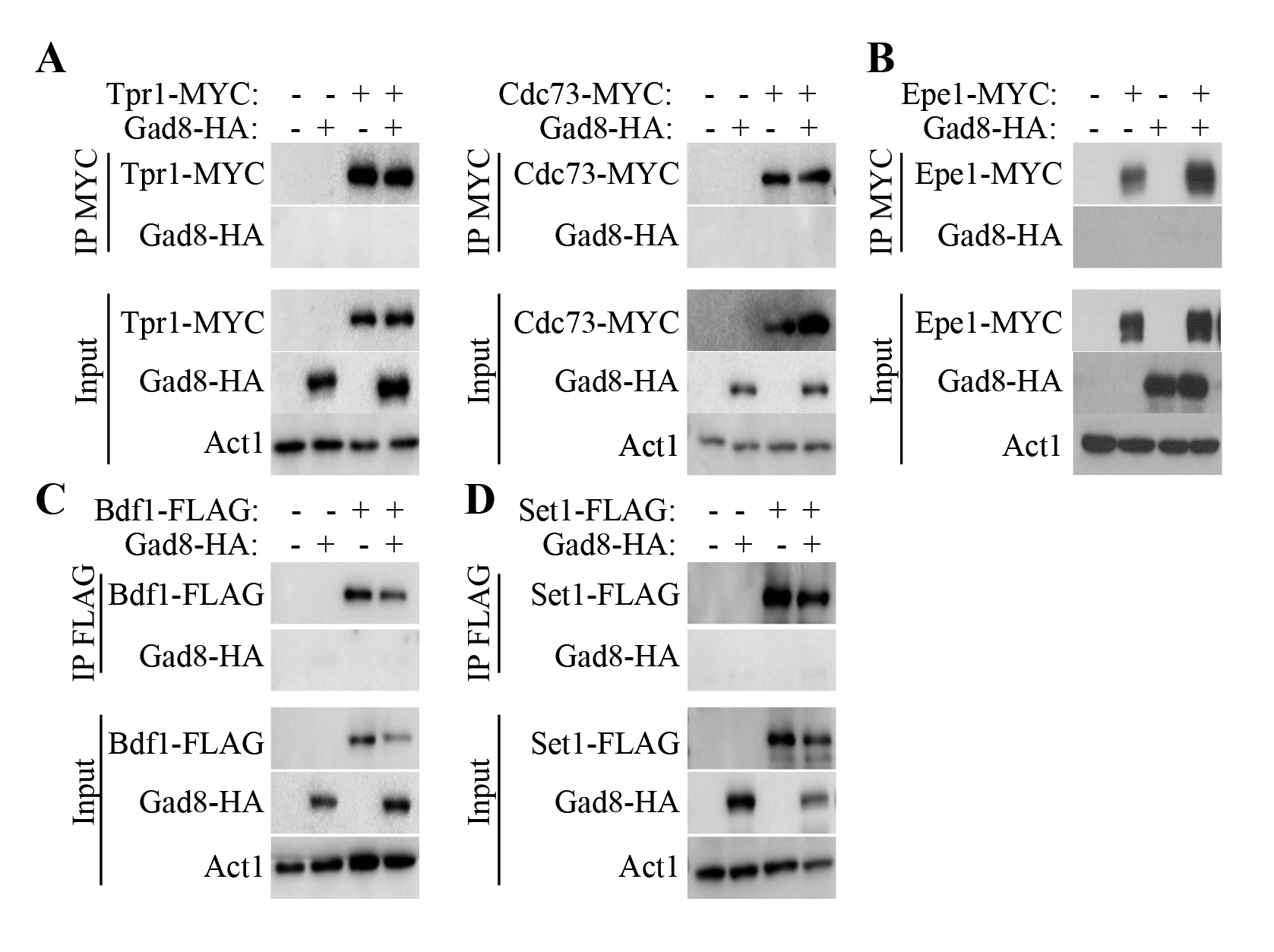

Supplement: S1 Fig — Gad8 does not associate with members of the Paf1C complex, Tpr1 and Cdc73 (A), Epe1 (B), Bdf1 (C) and Set1 (D). Protein extracts from wild type cells expressing the indicated chromosomally tagged proteins were immunoprecipitated (IP) with anti-MYC or anti-FLAG antibody. Western blots were performed using either anti-HA, anti-FLAG or anti-MYC to detect the presence of tagged proteins within the immune complexes. The expression levels of the indicated proteins before IP is shown (Input). Act1 was used as a loading control. (TIF) [file pgen.1010061.s001.tif]

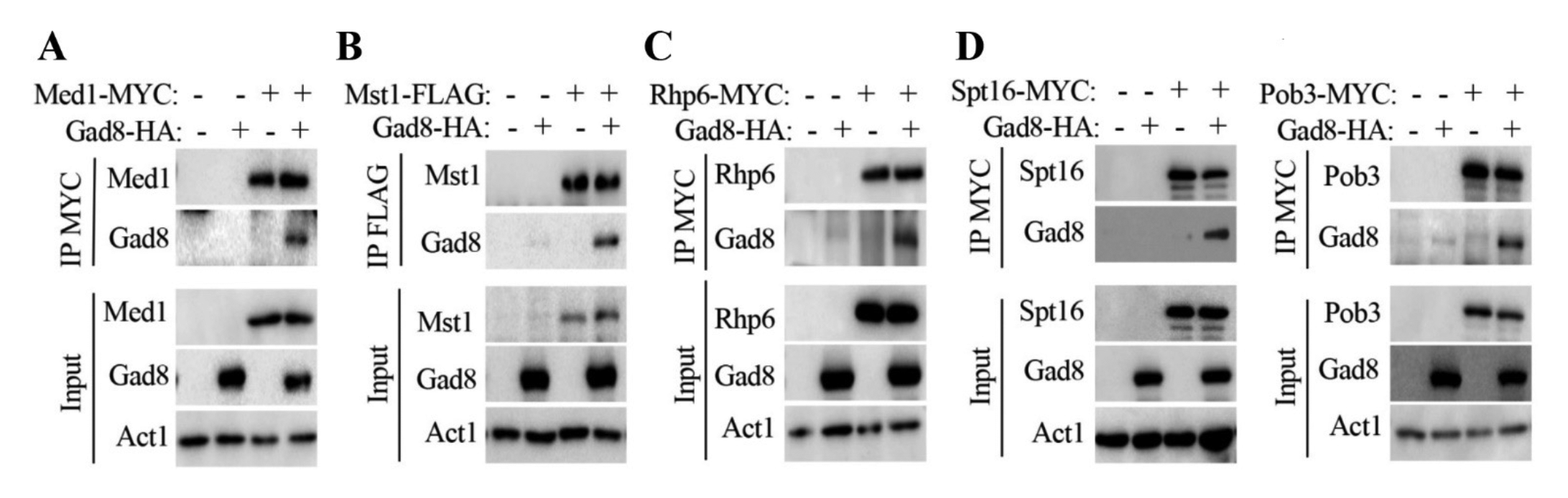

Supplement: S2 Fig — Gad8 associates with Med1 (A), Mst1 (B), Rhp6 (C) and the subunits of the FACT complex, Spt16 and Pob3 (D). Protein extracts from wild type cells expressing the indicated chromosomally tagged proteins were immunoprecipitated (IP) with anti-MYC or anti-FLAG antibody. Western blots were performed using either anti-HA, anti-FLAG or anti-MYC to detect the presence of tagged proteins within the immune complexes. The expression levels of the indicated proteins before IP is shown (Input). Act1 was used as a loading control. (TIF) [file pgen.1010061.s002.tif]

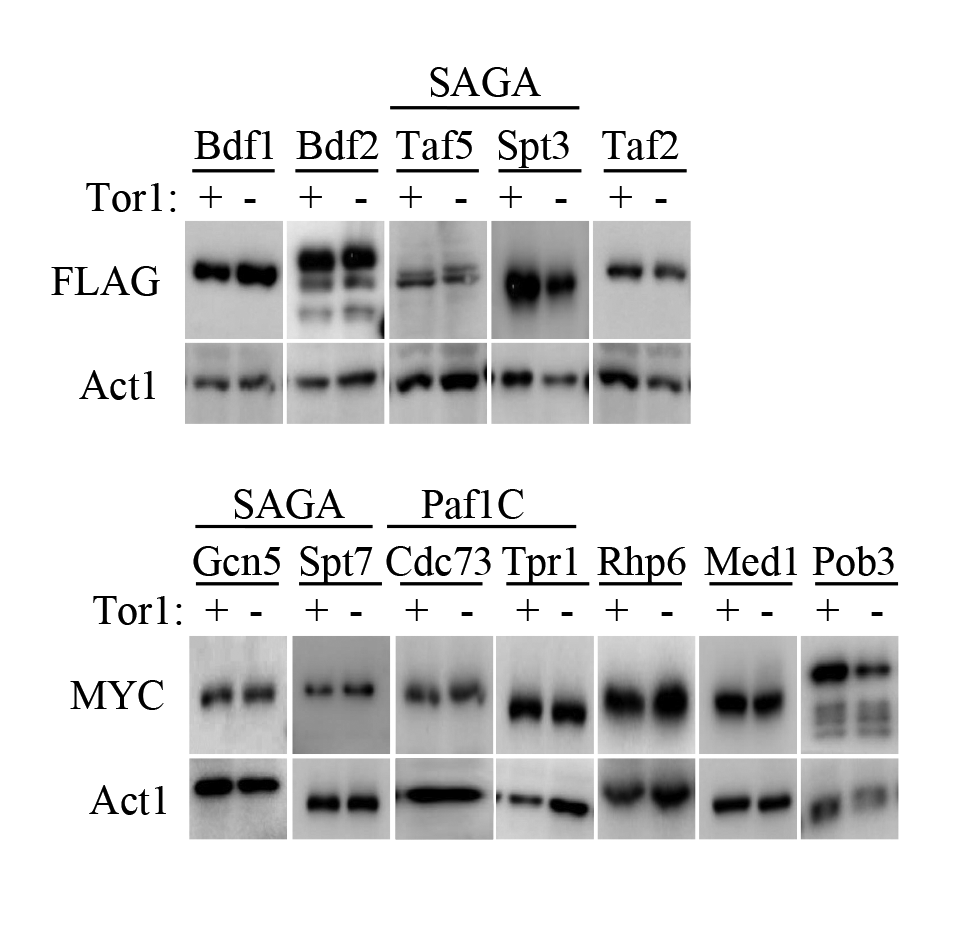

Supplement: S3 Fig — Wild type cells expressing the indicated chromosomally tagged proteins were grown to mid-logarithmic phase. The proteins were extracted with TCA and loaded on Phos-tag gels. Western blot analyses were performed using either anti-MYC or anti-FLAG. Act1 was used as a loading control. We detected mobility shifts for several proteins (Bdf2, Taf5 and Pob3), suggesting that these proteins are subjected for phosphorylation, however, no differences were observed in Δtor1 cells. (TIF) [file pgen.1010061.s003.tif]

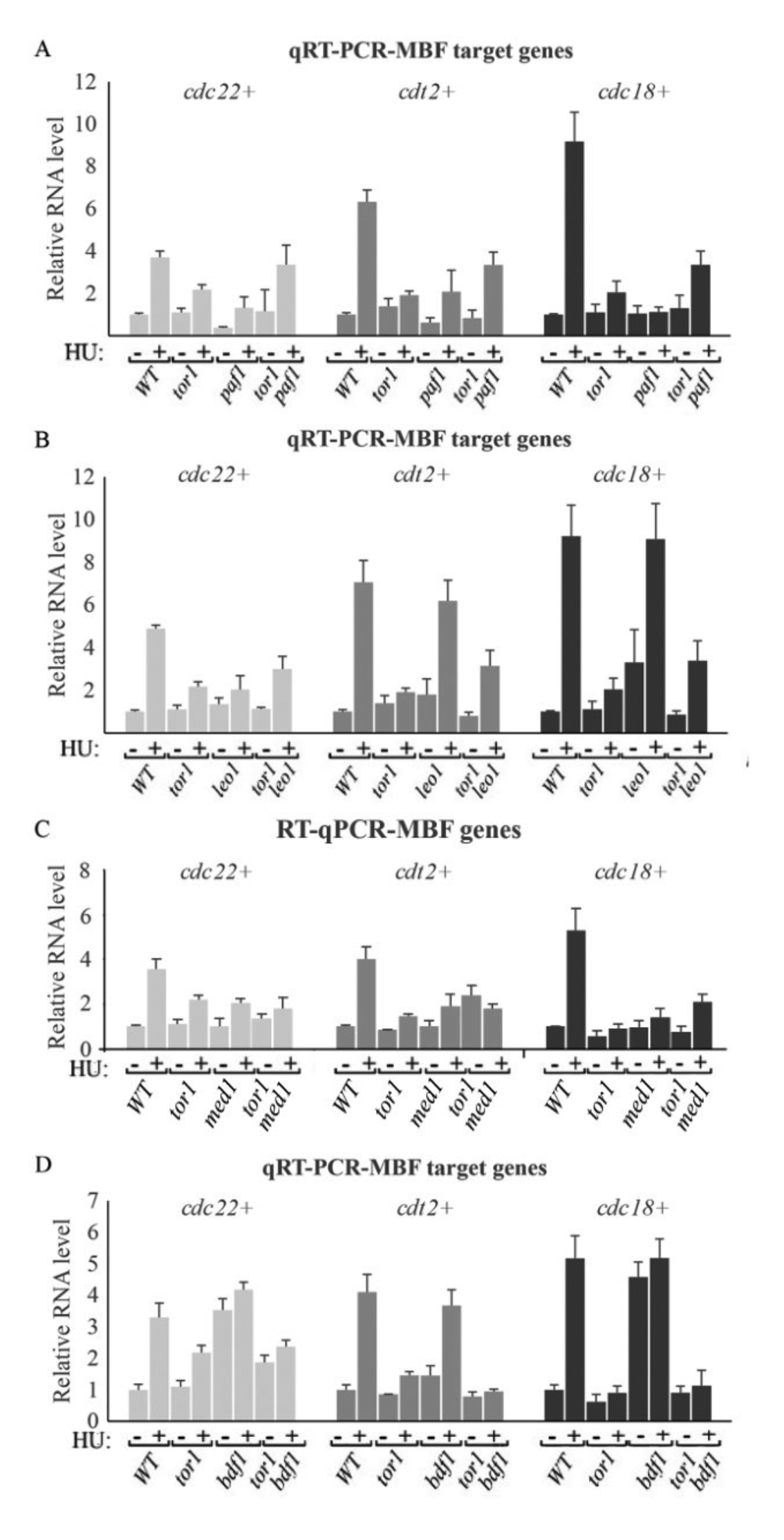

Supplement: S4 Fig — (A-D) Expression levels of cdc22+, cdt2+ and cdc18+ in wild type (WT) and indicated deletion mutant cells were determined by RT-qPCR. Total RNA was prepared from untreated cells (-) or cells treated with 12 mM HU for 3 hours (+). The level of act1+ mRNA was used as a reference. Each value is the mean of at least three independent assays, and the error bars indicate standard deviation. (TIF) [file pgen.1010061.s004.tif]

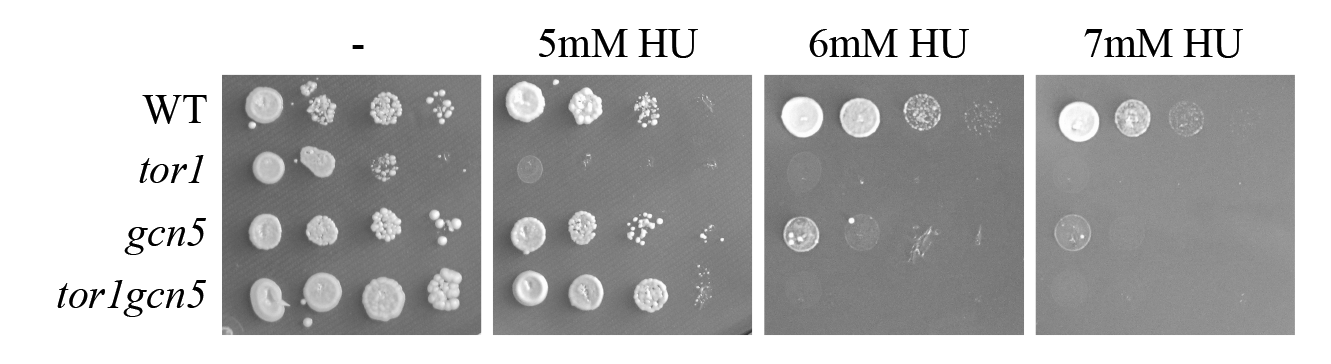

Supplement: S5 Fig — Serial dilution of exponentially growing wild type, Δtor1, Δgcn5 or Δtor1 Δgcn5 cells were spotted onto minimal medium, with or without 5, 6 or 7 mM hydroxyurea (HU). Δgcn5 cells are sensitive to 6 or 7 mM HU. The Δgcn5 mutation rescues the sensitivity of Δtor1 cells at 5 mM HU, but not at higher HU concentrations. (TIF) [file pgen.1010061.s005.tif]

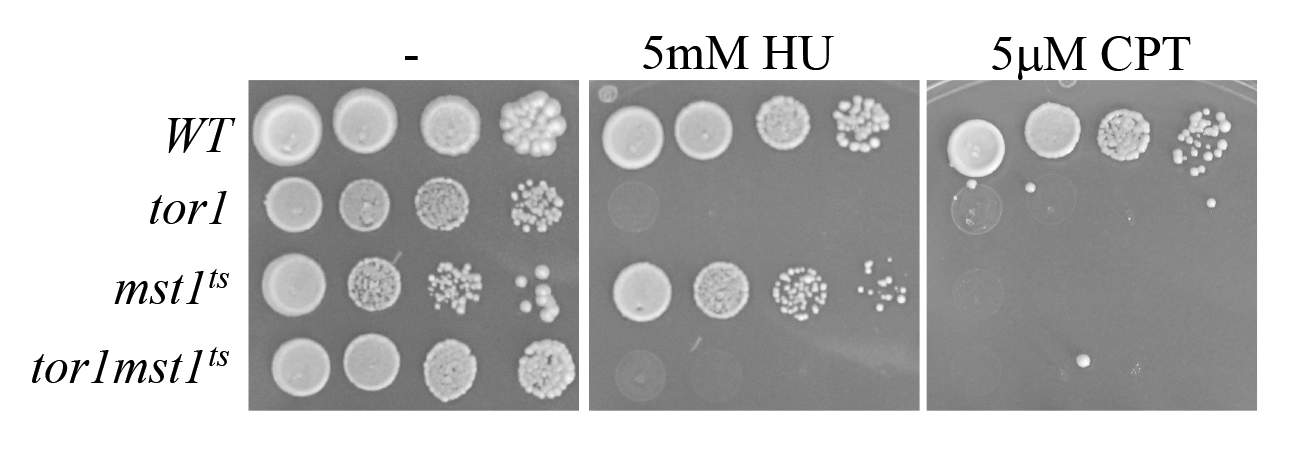

Supplement: S6 Fig — Serial dilution of exponentially growing wild type, Δtor1, mst1ts or Δtor1 mst1ts cells were spotted onto minimal medium, with or without 5 mM hydroxyurea (HU). The mst1ts strain is resistant to 5 mM HU, but sensitive to camptothecin (CPT). The mst1ts mutation does not rescue the sensitivity of Δtor1 to either HU or CPT. (TIF) [file pgen.1010061.s006.tif]

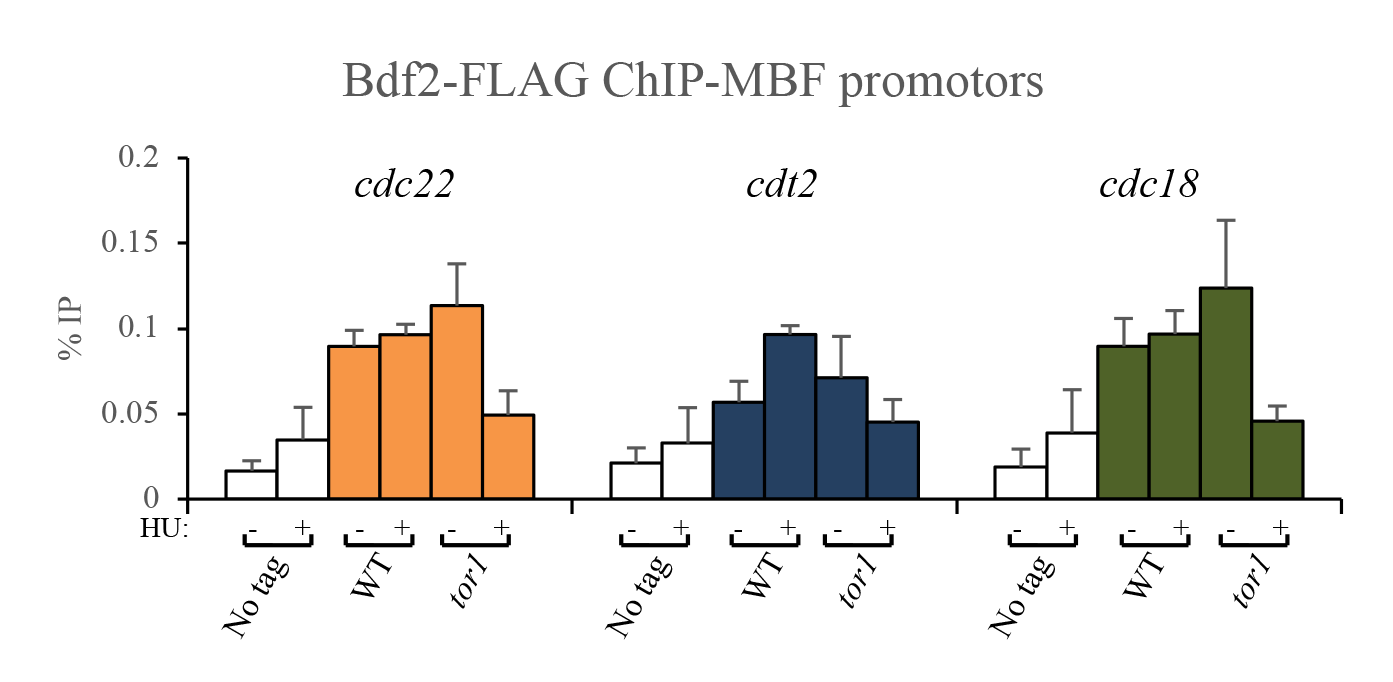

Supplement: S7 Fig — Loading of Bdf2 at the promoters of cdc22+, cdt2+ and cdc18+ was measured by chromatin immunoprecipitation (ChIP) analysis of chromatin extracts isolated from untagged cells (No tag), wild type cells carrying Bdf2-FLAG (WT) or Δtor1 cells carrying Bdf2-FLAG (tor1). Cells were untreated (-) or treated with 12 mM HU for 3 hours (+). The level of binding is quantified on anti-FLAG immunoprecipitated DNA by quantitative PCR. (TIF) [file pgen.1010061.s007.tif]

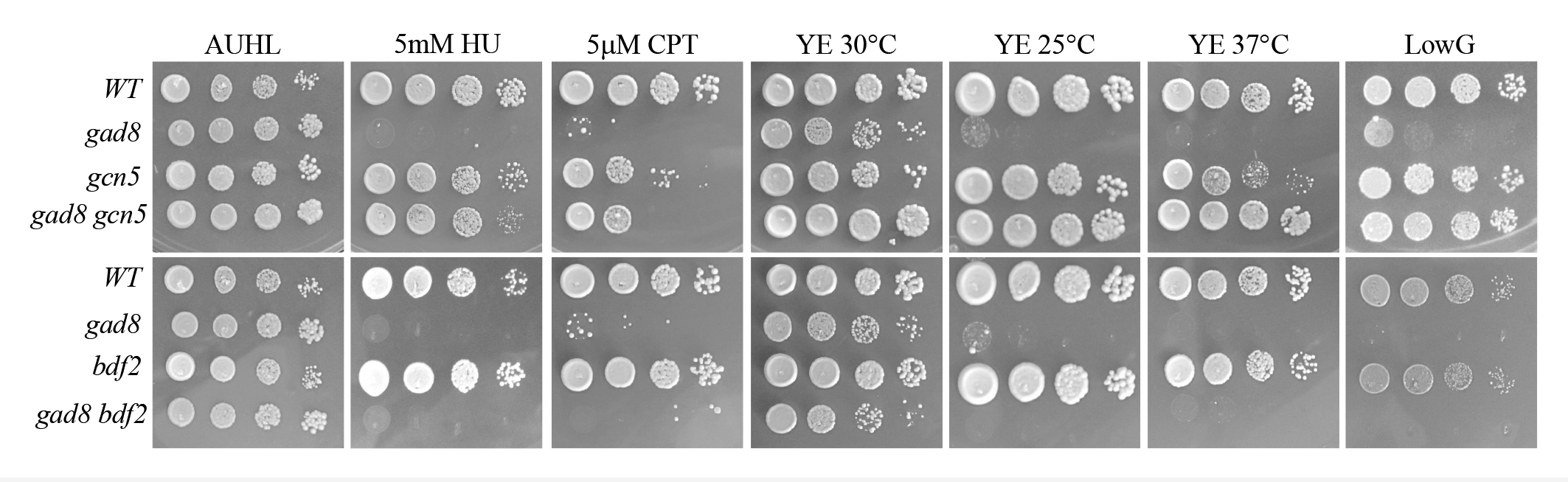

Supplement: S8 Fig — Serial dilution of exponentially growing wild type and deletion mutant cells were spotted onto minimal medium (EMM), with or without hydroxyurea (HU) camptothecin (CPT) or onto rich medium (YE) at standard temperature (30°C), low temperature (25°C), high temperature (37°C) or YE medium in which glucose was replaced with 2% galactose and 0.1% glucose (LowG). (TIF) [file pgen.1010061.s008.tif]
