# Supplementary material for: TOR complex 2 contributes to regulation of gene expression via inhibiting Gcn5 recruitment to subtelomeric and DNA replication stress genes
Source: PLoS Genet. 2022 Feb 14;18(2):e1010061. doi: 10.1371/journal.pgen.1010061 (PMC8880919; doi:10.1371/journal.pgen.1010061)
Supplement: S1 Table — (DOCX) [file pgen.1010061.s009.docx]

**S1 Table.** **Strains used in this study**

| **Strain** | **Genotype** | **Source** |
| --- | --- | --- |
| TA1 | *975 h^+^* | Laboratory stock |
| TA2 | *h^-^* *leu1-32 ura4-D18 ade6-M210* | Laboratory stock |
| TA3 | *h^+^* *leu1-32 ura4-D18 ade6-M210* | Laboratory stock |
| TA16 | *h^90^ leu1-32 ura4-D18 ade6-M216* | Laboratory stock |
| TA101 | *h^-^ tor1::ura4^+^ leu1-32 ura4-D18 ade6-M216* | Laboratory stock |
| TA390 | *h^-^ tor1::ura4^+^ ura4-D18* | Laboratory stock |
| TA1126 | *h^90^ gad8::ura4<<kanMX-gad8-6HA leu1-32 ade6-M210* | *YGRC |
| TA2528 | *h^90^ epe1-13MYC::kanMX6 leu1-32 ura4-D18 ade6-M216* | This study |
| TA2534 | *h^90^ gad8::ura4<<hphMX6-gad8-6HA leu1-32 ade6-M210* | This study |
| TA2560 | *h^90^ gad8::ura4<< hphMX6 -gad8-6HA epe1-13MYC::kanMX6 leu1-32 ura4-D18 ade6* | This study |
| TA2626 | *h^90^* *kanMX6-spt16-13MYC leu1-32 ura4-D18 ade6-M210* | This study |
| TA2635 | *kanMX6-spt16-13MYC gad8::ura4<< hphMX6 -gad8-6HA leu1-32 ura4-D18 ade6* | This study |
| TA2662 | *h^90^ kanMX6-gcn5-13MYC gad8::ura4<< hphMX6 -gad8-6HA leu1-32 ura-D18 ade6* | This study |
| TA2729 | *h^90^ gad8::ura4^+^ kanMX6-gcn5-13MYC leu1-32 ura4-D18 ade6-M216* | This study |
| TA2744 | *h^90^ tor1::ura4^+^ kanMX6-gcn5-13MYC leu1-32 ura4-D18 ade6-M216* | This study |
| TA2893 | *h^90^ gcn5::kanMX6 leu1-32 ura4-D18 ade6-M216* | This study |
| TA2947 | *h^90^ gad8::ura4^+^ gcn5::kanMX6 leu1-32 ura4-D18 ade6-M216* | This study |
| TA3073 | *h^90^ tor1::ura4^+^ paf1::KanMX6 leu1-32 ura4-D18 ade6* | This study |
| TA3430 | *h^90^ gad8::kanMX6 leu1-32 ura4-D18 ade6-M216* | This study |
| TA3852 | *leo1:: hphMX6* | This study |
| TA3875 | *tor1:: hphMX6 leo1:: kanMX6* | This study |
| TA3891 | *h^-^ bdf2::hphMX6 leu1-32 ura4-D18 ade6-M210* | This study |
| TA3892 | *h^-^ bdf1::hphMX6 leu1-32 ura4-D18 ade6-M210* | This study |
| TA3907 | *bdf2::hphMX6 tor1::ura4^+^leu1-32 ura4-D18 ade6* | This study |
| TA3908 | *bdf1::hphMX6 tor1::ura4^+^ leu1-32 ura4-D18 ade6* | This study |
| TA3990 | *tor1::ura4^+^ pob3-13Myc<kanMX6 leu1-32 ura4-D18 ade6-M216* | This study |
| TA3996 | *kanMX6-rhp6-13MYC gad8::ura4<< hphMX6 -gad8-6HA leu1-32 ura4-D18 ade6* | This study |
| TA4002 | *tor1::ura4^+^rhp6-13MYC::kanMX6 leu1-32 ura4-D18 ade6-M216* | This study |
| TA4006 | *kanMX6-ubp8-13MYC gad8::ura4<< hphMX6-gad8-6HA leu1-32 ura4-D18 ade6* | This study |
| TA4025 | *h^90^ tor1::ura4^+^ gcn5::kanMX6 leu1-32 ura4-D18 ade6-M216* | This study |
| TA4037 | *h^+^ set1-5FLAG<hphMX6 leu1-32 ura4-D18 ade6-M210* | This study |
| TA4042 | *h^+^ hphMX6-leo1-5FLAG leu1-32 ura4-D18 ade6-M210* | This study |
| TA4044 | *set1-5FLAG<< hphMX6 gad8::ura4<<kanMX-gad8-6HA leu1-32 ura4-D18 ade6-M210* | This study |
| TA4062 | *hphMX6-leo1-5FLAG gad8::ura4<<kanMX-gad8-6HA leu1-32 ura4-D18 ade6* | This study |
| TA4063 | *hphMX6-paf1-5FLAG gad8::ura4<<kanMX-gad8-6HA leu1-32 ura4-D18 ade6* | This study |
| TA4115 | *h^-^* *kanMX6-med1-13MYC leu1-32 ura4-D18 ade6-M210* | This study |
| TA4142 | *kanMX6-med1-13MYC gad8::ura4<< hphMX6-gad8-6HA leu1-32 ura4-D18 ade6* | This study |
| TA4155 | *tor1::ura4^+^ med1-13Myc<kanMX6 leu1-32 ura4-D18 ade6-M216* | This study |
| TA4182 | *h^-^ tpr1-13MYC< kanMX6 leu1-32 ura4-D18 ade6-M210* | This study |
| TA4190 | *h^-^ med1::hphMX6 Leu1-32 ura4-D18 ade6-M210* | This study |
| TA4216 | *h^-^ cdc73-13MYC< kanMX6 leu1-32 ura4-D18 ade6-M210* | This study |
| TA4219 | *med1::hphMX6  tor1::ura4^+^ leu1-32 ura4-D18 ade6* | This study |
| TA4220 | *gad8::ura4<<hph-gad8-6HA tpr1-13MYC::kanMX6 leu1-32 ura4-D18 ade6* | This study |
| TA4231 | *gad8::ura4<< hphMX6-gad8-6HA Cdc73-13MYC::kanMX6 leu1-32 ura4-D18 ade6* | This study |
| TA4236 | *tor1::ura4^+^ tpr1-13Myc<kanMX6 leu1-32 ura4-D18 ade6-M216* | This study |
| TA4240 | *tor1::ura4^+^ spt7-13Myc<kanMX6 leu1-32 ura4-D18 ade6-M216* | This study |
| TA4444 | *tor1::ura4^+^ cdc73-13MYC::kanMX6 leu1-32 ura4-D18 ade6-M216* | This study |
| TA4544 | *h^-^ tor1::ura4^+^ubp8::hphMX6* | This study |
| TA4562 | *h^-^ mst2:: hphMX6 leu1-32 ura4-D18 ade6-M210* | This study |
| TA4572 | *tor1::ura4^+^ mst2:: hphMX6 leu1-32 ura4-D18 ade6 his2* | This study |
| TA4640 | *h^+^ hphMX6-bdf2-5FLAG leu1-32 ura4-D18 ade6-M210* | This study |
| TA4641 | *hphMX6-bdf2-5FLAG gad8::ura4<<kanMX-gad8-6HA leu1-32 ura4-D18 ade6* | This study |
| TA4642 | *h^+^ hphMX6-spt3-5FLAG leu1-32 ura4-D18 ade6-M210 his1-102* | This study |
| TA4646 | *h^+^* *hphMX6-mst1-5FLAG leu1-32 ura4-D18 ade6-M210* | This study |
| TA4648 | *hphMX6-spt3-5FLAG gad8::ura4<<kanMX-gad8-6HA leu1-32 ura4-D18 ade6-M210* | This study |
| TA4651 | *tor1::ura4^+^ Spt3-5FLAG<kanMX6 leu1-32 ura4-D18 ade6-M216* | This study |
| TA4655 | *hphMX6-mst1-5FLAG gad8::ura4<<kanMX-gad8-6HA leu1-32 ura4-D18 ade6* | This study |
| TA4660 | *tor1::ura4^+^ hphMX6-bdf2-5FLAG leu1-32 ura4-D18 ade6-M216* | This study |
| TA4679 | *h^+^ hphMX6-taf5-5FLAG leu1-32 ura4-D18 ade6-M210* | This study |
| TA4681 | *hphMX6-taf5-5FLAG gad8::ura4<<kanMX-gad8-6HA leu1-32 ura4-D18 ade6* | This study |
| TA4682 | *h^+^* *hphMX6-taf2-5FLAG leu1-32 ura4-D18 ade6-M210* | This study |
| TA4686 | *tor1::ura4^+^ taf5-5FLAG<hphMX6 leu1-32 ura4-D18 ade6-M216* | This study |
| TA4690 | *hphMX6-taf2-5FLAG gad8::ura4<<kanMX-gad8-6HA leu1-32 ura4-D18 ade6* | This study |
| TA4701 | *tor1::ura4^+^ taf2-5FLAG<hphMX6 leu1-32 ura4-D18 ade6-M216* | This study |
| TA4721 | *h^+^ bdf1-5FLAG::hphMX6 leu1-32 ura4-D18 ade6-M210* | This study |
| TA4729 | *bdf1-5FLAG< hphMX6 gad8::ura4<<kanMX-gad8-6HA leu1-32 ura4-D18 ade6-M210* | This study |
| TA4746 | *tor1::ura4^+^ bdf1-5FLAG<hphMX6 leu1-32 ura4-D18 ade6-M216* | This study |
| TA4757 | *h^-^ gcn5::hphMX6 leu1-32 ura4-D18 ade6-M210* | This study |
| TA4762 | *h^-^ set2::kanMX6 gcn5::hphMX6 leu1-32 ura4-D18 ade6-M210* | R. Allshire lab |
| TA4774 | *tor1::ura4^+^ set2::kanMX6 gcn5::hphMX6 ade6-M210 leu1-32 ura4-D18* | This study |
| TA4778 | *tor1::ura4^+^ bdf2::HphMX6 kanMX6-gcn5-13MYC leu1-32 ura4-D18 ade6* | This study |
| TA4815 | *h^-^ mst1-L344S-5FLAG:kanMX6 leu1-32 ura4-D18* | E. Noguchi lab |
| TA4835 | *h^-^ tor1::ura4^+^mst1-L344S-5FLAG:kanMX6 leu1-32 ura4-D18* | This study |
| TA4897 | *h^90^ bdf2::kanMX6 leu1-32 ura4-D18 ade6-M216* | This study |
| TA4956 | *tor1::ura4^+^ gcn5::hphMX6 Leu1-32 ura4-D18 ade6-M210* | This study |
| TA4990 | *gcn5::kanMX6  mst2::hphMX6* *leu1-32 his2 ura4-D18 ade6* | This study |
| TA5007 | *tor1::ura4^+^ gcn5::kanMX6 mst2::hphMX6 leu1-32 ura4-D18 ade6 his2* | This study |
| TA5060 | *h^90^ bdf2::hphMX6 gad8:: kanMX6 leu1-32 ura4-D18 ade6* | This study |

*YGRC Yeast Genetic Resource Center, Japan
