# Supplementary material for: TOR complex 2 contributes to regulation of gene expression via inhibiting Gcn5 recruitment to subtelomeric and DNA replication stress genes
Source: PLoS Genet. 2022 Feb 14;18(2):e1010061. doi: 10.1371/journal.pgen.1010061 (PMC8880919; doi:10.1371/journal.pgen.1010061)
Supplement: S2 Table — (DOCX) [file pgen.1010061.s010.docx]

**S2 Table. Oligonucleotides used for gene deletions and protein tagging**

| Name | | Sequence |
| --- | --- | --- |
| *paf1::kanMX4* | #1593 F | ACGCTCGTTTCCAATTTAGGGTGATGCTAGGAAGTTATATGGGAACGTGATCTCCTTGGGTATTTGTGGAAAACACATCACGGATCCCCGGGTTAATTAA |
|  | #1594 R | TACAAGGGTATGCTGCATGAAAGACACGATTAGATCATTTATTTGTTTAGGATAATGTTTCATTTTCAAACATGAAACGAGAATTCGAGCTCGTTTAAAC |
| *Paf1-5FLAG* | #1683 F | AAGCAAATTCCCCTGCACCACCAGTAGAAGAAGGGAATACACAGCCTTCGCCAGTTGAACAGCTACAAAATGAAGAGGATCGGATCCCCGGGTTAATTAA |
| *leo1::kanMX4* | #1596 F | TACAACACCATACGTATTCTGACCGGAACCAATCTGGAAAGTTTAGACTGGTGAATTTTTGAGGAATTTAAATAGATTTACGGATCCCCGGGTTAATTAA |
|  | #1597 R | CAACGAAATAACACAGAGACCATACTTCGCAAACAAACAAATTGGCTATTTACAAAAATTGAATTACAAGCTGTTTGGATGAATTCGAGCTCGTTTAAAC |
| *Leo1-5FLAG* | #1684 F | AGACCGTTACAGAGACTTCTGTCGGTGATGGTTCTGTACAAAGAAGAGTGAAGCGTCGGATTGTTGAGAGCGATAGCGAACGGATCCCCGGGTTAATTAA |
| *bdf2::hphMX4* | #1630 F | GACTACTTTCGGTTGTGCCCGAACCTTGAATTTTGAATCTACCCTTCGCCAACCTCAAAATTGCTGGATTGTACCAGAATCGGATCCCCGGGTTAATTAA |
|  | #1631 R | TGAAGTGAGAGGTTACAAAAACATTAAACATTAAAAATAAAAAGGCACCAAAGGATACCTCTCATCTAAGGAAGTATTAAGAATTCGAGCTCGTTTAAAC |
| *Bdf2-5FLAG* | #1735 F | CTTATAATACTAAATCGTTAGGCAGTGATGATTCTTCCTCTGAAGATGATGGTGAAAGCAGTGAAAGTAGCGATTCCGCACGGATCCCCGGGTTAATTAA |
| *gcn5::kanMX4* | #1216 F | TTCATCTTGTATCGTTCTTGACAATTTCTGTATCTTCACTTTTTGGATTTATTTGTTTGGATGCGTGGCATAGAATAATCCGGATCCCCGGGTTAATTAA |
|  | #1123 R | AAAAATTAAAAGGTGAAATGTATATGTTATAATCAATAAAACTTCGGAATAGACGTTTCGATGATAATAAATGTAAATGAGAATTCGAGCTCGTTTAAAC |
| *Gcn5-5FLAG* | #1122 F | CTTACTATAAAAATGCCGATAGATTGGAAAAGTTTTTCCAGAAAAAACTTCGTGAAACTGAGTATTCACACTTAGCCGATCGGATCCCCGGGTTAATTAA |
| *ubp8::hphMX4* | #1646 F | GTTCGTCTATTGTTGTCGTTTCACCCTGCATCGAAGCAGATGCTTTTTAGTCTGAAAGGCCAATTTTGTACGTATCAAGGCGGATCCCCGGGTTAATTAA |
|  | #1647 R | AATAAGGAACCTTTGAGTCAATTACTTTACATGGTTCATAATGATTGTTCATGAACGGATATCTGATCGCATAATCTAACGAATTCGAGCTCGTTTAAAC |
| *Ubp8-5FLAG* | #1675 F | TAAATTCTCAAGCTTACCTATTATTTTACCATGAGCGTCAAATCCTATACAGTGATGAAATGACTGTTAAAACGGAAAATCGGATCCCCGGGTTAATTAA |
| *Spt7-13MYC* | #1732 F | TTTTAAATCAATCCTTGAGAAAAAAGCGCTGCCTAAAGGAGAATGAGCAAGGTACTGAGGTAACTACTCTTCCTGAAGAACGGATCCCCGGGTTAATTAA |
|  | #1731 R | TTTAAAAGTTATGTCTCCATTGTGGTTGATACACATCTATATACTAGTTGTTTTTGACGTTATAAATAAATACATATGGCGAATTCGAGCTCGTTTAAAC |
| *Taf5-5FLAG* | #1764 F | TAGCTCTTTATACGAAGCAAACCCCTATATTTAATGTCTCCTTTACGCGTCGTAACCTTTGCTTAGCAATAAGCGTTAGTCGGATCCCCGGGTTAATTAA |
|  | #1763 R | AATCTTTTTTTATATAATGCCAAATTTCTTAAAGTATGGACGGGAAGAAACATATTGAAGATCAAAAAAGGTTTATTTATGAATTCGAGCTCGTTTAAAC |
| *Spt3-5FLAG* | #1712 F | GTTTGCAAATCCCCGATAAAACTCATACTGCTATGCGCAGTTTTCATGGGGGTTTGGTCAAATCTCGTGTGTATCTTATCCGGATCCCCGGGTTAATTAA |
|  | #1711 R | TAATTACAGGAAAACAGGGCATTTAGATGTAAATGGAAGCCTTACAGCAATTAAAAAAAAAGAAGAGACAGTGAAAATGAGAATTCGAGCTCGTTTAAAC |
| *Rhp6-13MYC* | #1655 F | GGTGTAATTCCAAGGCGATATCGATATTTGTGCAACTTTTTTTTAAAGTTATCACAAATAGAAGAGAGGTTGCTATAAAACGGATCCCCGGGTTAATTAA |
|  | #1656 R | AGATTTAATGTGAAAGGCGGTTGAAAAAGAAGAGTAAGTTCTAATGAAATAGGGATTATTAAGACAGCTATGTCTTGAAAGAATTCGAGCTCGTTTAAAC |
| *Mst1-5FLAG* | #1090 F | AAAGAAGAAGGATCAATGGAGATTTGTTGGCAGATTGGCAACCTCCTGTTTTTCACCCTTCCCAACTACGATTTGGATGGCGGATCCCCGGGTTAATTAA |
|  | #1089 R | TCTAGGAGATACTTTAATTATGAGAATTTTTTTTTTTTTTAAGAAAGACGACCAGCATCTAATCCATTCTTTGTACAGAAGAATTCGAGCTCGTTTAAAC |
| *Pob3-13MYC* | #1674 F | AAAGCGATGTTGCTGAAGAATATGATGAAAATGCAGAATCTTCTGATGAAGAAGGTGCTTCTGGTGCTGAGGGTTCGGAGCGGATCCCCGGGTTAATTAA |
|  | #1625 R | AATTTGTAATAGCAAAAGACAAGGCTGAAAAGTTGATCCAAATAGACTTCCAATTTAACATTGCTCAATTTTATAGATCAGAATTCGAGCTCGTTTAAAC |
| *Spt16-13MYC* | #1105 F | ATGAATTGGAGCGTAAAGCACGTCAAGAAGATGCTAAGCACGATGCTTTTGAAGAAAGACCATCTAAAAAGAGACACCGGCGGATCCCCGGGTTAATTAA |
|  | #1106 R | AATATTTTATTATAAAGTATTCTGGAATTTTTAGAATAAACTACATATAAATAATAAGAAAGTGAAGTAAATTTGTGAGTGAATTCGAGCTCGTTTAAAC |
| *med1::hphMX4* | #1699 F | GCGCTTATATACAGTGTTTTAAGCTGATTAAATACGAAATACACGACTAATTTACTAAATCTTCTTTAATAATCTTTTCGCGGATCCCCGGGTTAATTAA |
|  | #1700 R | AACATCCAACTAATTCCATAATTTCATTTTACTAAAGATTTTTAAAACTGATTAGTAATGTCTTTTTCTAGCATGAAATTGAATTCGAGCTCGTTTAAAC |
| *Med1-13MYC* | #1701 F | CTCATGTTTTTCAAAAACTTATACAACAAACATGTAACATCGGACTTGCGATAGAAGTGTTTATAAAGAAAGTCGTTAATCGGATCCCCGGGTTAATTAA |
| *Taf2-5FLAG* | #1786 F | TAACAATCAATCCTATTAAACCAAAGGGACCTACTTTGAAAATAAAGCTAACAAATCTCCGCTCTACTCCACCTTCTCATCGGATCCCCGGGTTAATTAA |
|  | #1785 R | TAGATAGTGGCAGAGAATATGCAAAGTGTTAGCCAGAACTTAATCTGTTGTTTCTCATCCAACATTTTCGTCAACTTAACGAATTCGAGCTCGTTTAAAC |
| *bdf1::hphMX4* | #1627 F | ATGTTAATATTAACTTTTTTAGGGTGAAGGAGGCGTTCACAGATATTATTACTGTCCTCAATAAGCTTGAAGATCTCGATCGGATCCCCGGGTTAATTAA |
|  | #1628 R | AGGAAAGATAAAAAAGGGGAACAATAAAAAGAGCCTTAAAAAAATAAATCAAAGAATTTGGTTAAACCTGCTAACCACATGAATTCGAGCTCGTTTAAAC |
| *mst2::hphMX4* | #1802 F | TTGACCAAAGGATTAAATGGGGCCATTAGGAAAACTATGAACGATCTGTAAATATAAACAATCTTTTTTTTTTATGTATACGGATCCCCGGGTTAATTAA |
|  | #1803 R | TATAGAGCAACAACCAAGCCGTAGATGATACAAATGCTTCACGACAAATATCGAAAGATTAAAATACTTATTTATTTGAAGAATTCGAGCTCGTTTAAAC |
| *gad8:: kanMX4* | #682 F | TTAAAAGAAAAGATAGAGGGAAAGCGAGCTTTTAAAAATCAGTTCATTTTTTTTTTCTACTCCAAACAGACGTTACCGAACGGATCCCCGGGTTAATTAA |
|  | #683 R | ATGTAAAAGAGGCAAGAAAAGCGGCATGTATGAGTAAAAATGAGAAAACTTTCAAAATAAACAAAGAAGTGTCAAATTCAACATTTGATTAAAATAGAAC |
| *Tpr1-5FLAG* | #1716 F | AAGAAAAGCAGGATAACGATATCACAGACAATCAAGATGACAATAAAGAGTTAAATCTATTCTCTGAAGAGGATGAAGAACGGATCCCCGGGTTAATTAA |
|  | #1715 R | AAGGGAGAAAGTTAATATGCCATTGAAATAAGAAAATTCTTATCTGAACGGCATAATTCAAACAGCAATGAGCATAATTAGAATTCGAGCTCGTTTAAAC |
| *Cdc73-5FLAG* | #1720 F | ATCGTGAAGTAGTCAGTCAATTGTGGGATAAGTTAGAACGCTGGATGGAAAATAGATGGCCGCTTTGGAACGGCAGAAGGCGGATCCCCGGGTTAATTAA |
|  | #1719 R | ATAAATTCATTAAGAAAGTGAAAAATAGTTTTTTAGCAGTAATTATAAAGAACGGGATTTGAAAACATTTATATACATAAGAATTCGAGCTCGTTTAAAC |
| *Epe1-5FLAG* | #1097 F | AAAATAACATTTATGATTTTGAAGATCACTCTCCTGTTAGGGAAAAATGGGGGCACAGGCTTCGGTCCAGAGGTGCTAGTCGGATCCCCGGGTTAATTAA |
|  | #1096 R | TGTGAACTACTCAAGAATCATAAGCACGTGGGGATAAATATTCAATGGTAGCCGAAGGAAATAAAAAGTGCCGAGGTACTGAATTCGAGCTCGTTTAAAC |
| *Bdf1-5FLAG* | #1734 F | CCACTTCTCCCGAGTCAAATAACGCTGCTAATGTTTCCGATTCTGAAAGTGACAATGAAAGTGAAAGTAGTGAATCTGCTCGGATCCCCGGGTTAATTAA |
|  | #1631 R | TGAAGTGAGAGGTTACAAAAACATTAAACATTAAAAATAAAAAGGCACCAAAGGATACCTCTCATCTAAGGAAGTATTAAGAATTCGAGCTCGTTTAAAC |
| *Set1-5FLAG* | #1689 F | CTTATGATTACAAGTTTCCGGAAGAAGCTGATAAGATTCCTTGTTTGTGTGGTGCTCCAACATGTCGTGGCTATTTAAACCGGATCCCCGGGTTAATTAA |
|  | #1688 R | CGTGCTTTTTAAACGAACTATTATAATTGTACAGCTGCCCATATATTCATGTACCATCAATTCTCCAAGAGCATCATTGTGAATTCGAGCTCGTTTAAAC |
