# Supplementary material for: TOR complex 2 contributes to regulation of gene expression via inhibiting Gcn5 recruitment to subtelomeric and DNA replication stress genes
Source: PLoS Genet. 2022 Feb 14;18(2):e1010061. doi: 10.1371/journal.pgen.1010061 (PMC8880919; doi:10.1371/journal.pgen.1010061)
Supplement: S3 Table — (DOCX) [file pgen.1010061.s011.docx]

**S3 Table. Oligonucleotides used for qRT-PCR analyses**

| Name | | Sequence |
| --- | --- | --- |
| *Cdc22^+^* | #1128 F | ACTTAAAGTTCGGATGACGCGACG |
|  | #1129 R | GTTTGTAAGGTGGTAAATACCGGG |
| *Cdt2^+^* | #1130 F | CTGGGTAACGTTTGGTGCATGTGA |
|  | #1131 R | TGAGGTCGTGTGTTCCAGTT |
| *Cdc18^+^* | #1132 F | GGCATTTCATATCTTTGAGGATGAGTCGT |
|  | #1133 R | ATGTCGCGTTCAACTCTACGTGTC |
| *Spac186.04^+^* | #1271 F | GCGAAGAAAACCCAACAAGC |
|  | #1272 R | TCATCGTTTACTCTGATCCGTGA |
| *Spac186.05^+^* | #1242 F | AAATTTTCCCGGGCTTTCAT |
|  | #1243 R | TCCGACAATCACCGCTACC |
| *Spac186.06^+^* | #1273 F | GGGAGTGGAGCTGGATCAGT |
|  | #1274 R | CGCCACCAACATGAATATCG |
