# Supplementary material for: TOR complex 2 contributes to regulation of gene expression via inhibiting Gcn5 recruitment to subtelomeric and DNA replication stress genes
Source: PLoS Genet. 2022 Feb 14;18(2):e1010061. doi: 10.1371/journal.pgen.1010061 (PMC8880919; doi:10.1371/journal.pgen.1010061)
Supplement: S4 Table — (DOCX) [file pgen.1010061.s012.docx]

**S4 Table. Oligonucleotides used for ChIP analyses**

| Name | | Sequence |
| --- | --- | --- |
| *Cdc22^+^* | #1212 F | TGCAACGTGTTGAACGTAACGAGC |
|  | #1213 R | AGGTAATGAACGACGACCACGGTT |
| *Cdt2^+^* | #1210 F | TGAGACTGGAGCTCTTGAGCTGTT |
|  | #1211 R | TAGCATTGTTGTGAGCAAGCCAGC |
| *Cdc18^+^* | #1214 F | GTAGGCATGCAATTGAACTTGCGG |
|  | #1215 R | TCATAGCAGATGTCGCTCGGACAA |
| *Spac186.04^+^* | #1271 F | GCGAAGAAAACCCAACAAGC |
|  | #1272 R | TCATCGTTTACTCTGATCCGTGA |
| *Spac186.05^+^* | #1242 F | AAATTTTCCCGGGCTTTCAT |
|  | #1243 R | TCCGACAATCACCGCTACC |
